# Establishment of a murine culture system for modeling the temporal progression of cranial and trunk neural crest cell differentiation

**DOI:** 10.1242/dmm.035097

**Published:** 2018-12-12

**Authors:** Maria R. Replogle, Virinchipuram S. Sreevidya, Vivian M. Lee, Michael D. Laiosa, Kurt R. Svoboda, Ava J. Udvadia

**Affiliations:** 1 Department of Biological Sciences, University of Wisconsin-Milwaukee, Milwaukee, WI 53201, USA; 2 Joseph J. Zilber School of Public Health, University of Wisconsin-Milwaukee, Milwaukee, WI 53201, USA; 3 STEMCELL Technologies, Vancouver, BC V6A 1BC, Canada

**Keywords:** Chondrogenic differentiation, Differentiation timeline, Glial differentiation, Neuronal differentiation, Sox9, *In vitro* model of neural crest differentiation

## Abstract

The neural crest (NC) is a transient population of embryonic progenitors that are implicated in a diverse range of congenital birth defects and pediatric syndromes. The broad spectrum of NC-related disorders can be attributed to the wide variety of differentiated cell types arising from the NC. *In vitro* models of NC development provide a powerful platform for testing the relative contributions of intrinsic and extrinsic factors mediating NC differentiation under normal and pathogenic conditions. Although differentiation is a dynamic process that unfolds over time, currently, there is no well-defined chronology that characterizes the *in vitro* progression of NC differentiation towards specific cell fates. In this study, we have optimized culture conditions for expansion of primary murine NC cells that give rise to both ectodermal and mesoectodermal derivatives, even after multiple passages. Significantly, we have delineated highly reproducible timelines that include distinct intermediate stages for lineage-specific NC differentiation *in vitro*. In addition, isolating both cranial and trunk NC cells from the same embryos enabled us to make direct comparisons between the two cell populations over the course of differentiation. Our results define characteristic changes in cell morphology and behavior that track the temporal progression of NC cells as they differentiate along the neuronal, glial and chondrogenic lineages *in vitro*. These benchmarks constitute a chronological baseline for assessing how genetic or environmental disruptions may facilitate or impede NC differentiation. Introducing a temporal dimension substantially increases the power of this platform for screening drugs or chemicals for developmental toxicity or therapeutic potential.

This article has an associated First Person interview with the first author of the paper.

## Abstract (precis)

**Summary:** A novel method for isolating and expanding primary neural crest cells, and establishment of reproducible temporal benchmarks of differentiation, provides a potential screening platform for developmental toxicity or therapeutic capacity.

## INTRODUCTION

The neural crest (NC) comprises a transient, multipotent, embryonic progenitor cell population that uniquely contributes to a wide variety of tissues and structures in vertebrate animals. Specified at the borders of the neural plate, NC cells delaminate from the neuroepithelium at the dorsal aspect of the neural tube and become migratory. During this time, studies have shown that a majority of the NC cells remain multipotent *in vivo* (Baggiolini et al., 2015; Bronner-Fraser and Fraser, 1989, 1988; McKinney et al., 2013; Serbedzija et al., 1990). The eventual acquisition of specific cell fate is directed by differences in spatiotemporal patterning during development. Some of the factors influencing NC cell differentiation *in vivo* include the site of origination along the anterior-posterior neuraxis, the timing of emigration, the migratory pathway and the final sites of arrest within the embryo (Betancur et al., 2010; Bhatt et al., 2013; Simões-Costa and Bronner, 2015). The remarkable plasticity of the NC allows it to give rise to cell types as diverse in structure and function as neurons of the peripheral nervous system and cartilage-matrix-producing cells found within cephalic structures (Le Douarin, 1982). Given their broad contribution to a range of tissues, disruption at any stage of NC cell development can result in pleiotropic structural and functional anomalies (Bolande, 1997). Thus, understanding the molecular mechanisms that regulate the normal growth and differentiation of the NC is necessary for fully comprehending the etiology underlying a plethora of birth defects.

The ability to manipulate isolated NC cells *in vitro* is highly advantageous, particularly for the purpose of testing the relative contributions of intrinsic and extrinsic factors mediating self-renewal and differentiation. For example, *in vivo*, the cranial NC normally gives rise to differentiated cells of both ectodermal and mesoectodermal cell fates, while trunk NC produces mainly ectodermal derivatives. However, directed differentiation of trunk NC *in vitro* demonstrates that trunk NC cells have the capacity to give rise to both ectodermal and mesoectodermal cell types (Dupin et al., 2018). Direct comparisons of the two cell populations *in vitro* would allow us to tease apart any intrinsic differences in cell behavior or response to environmental cues. In addition, *in vitro* models of NC development are beneficial for elucidating protein-protein and protein-gene interactions that regulate the transcriptional programs underlying NC growth and differentiation along distinct lineages. In such studies, *in vitro* expansion of the NC is valuable as the number of NC cells per developing embryo is very low in comparison to the millions of cells needed to investigate molecular function using biochemical assays, such as co-immunoprecipitation and chromatin immunoprecipitation with sequencing (ChIP-seq) analysis.

Although methods for isolating, expanding and differentiating avian (Baroffio et al., 1991; Calloni et al., 2009; Cohen and Konigsberg, 1975; Etchevers, 2011; Kerosuo et al., 2015; Sieber-Blum and Cohen, 1980; Trentin et al., 2004) and rodent (Bixby et al., 2002; Etchevers, 2011; Ishii et al., 2012; Maurer et al., 2007; Pfaltzgraff et al., 2012; Stemple and Anderson, 1992) NC cells *in vitro* have been established, characterization of the cultured NC cells is limited to validation of cell identity and differentiation potential. However, reproducible landmarks that define the temporal progression of differentiation towards a particular cell fate *in vitro* have not previously been characterized. Because directed differentiation of NC cells *in vitro* occurs over several days, delineating temporal differences could enhance the experimental dynamic range for assessing the impacts of genetic or environmental manipulations of the cultured cells. Therefore, our aim was to establish a culture system of the NC that will enable future investigations assessing how genetic or environmental perturbations may facilitate or impede NC cell differentiation along various cell lineages.

Previous reports suggest a difficulty in long-term maintenance of murine NC cells in culture, prompting the development of murine NC cell lines. Two murine NC cell lines have previously been established (Ishii et al., 2012; Maurer et al., 2007). One cell line, O9-1, was clonally derived from cranial NC isolated from *Wnt1cre; R26R/EYFP* transgenic mouse embryos (Ishii et al., 2012). The second cell line, JoMa1, was established from clonally derived trunk NC immortalized with the oncogene *c-myc* (also known as *Myc*) (Maurer et al., 2007). Although established cell lines do overcome the obstacle of long-term maintenance, one inherent drawback is the phenotypic instability that occurs over time in culture (Geraghty et al., 2014; Ishii et al., 2012; Rao and Anderson, 1997). In addition, the O9-1 cells have diminished differentiation capacity as they lack the ability to give rise to neurons. Furthermore, it is not possible to directly compare cranial and trunk NC cell behavior using these cell lines because they were derived from different genetic backgrounds and are propagated under different conditions. Therefore, to conduct our temporal analysis of *in vitro* NC differentiation, we optimized conditions for propagating primary murine cultures of cranial and trunk NC cells.

Here, we have established methods for primary murine NC cell culture that maintains both self-renewal capabilities and broad differentiation potential over an extended period of time. Furthermore, we present a detailed characterization of cranial and trunk NC cells in culture, including the molecular and morphological changes that occur as the cells differentiate along the neuronal, glial and chondrogenic lineages over time. Specifically, we compared directed differentiation of cranial and trunk NC cells isolated from *Sox9cre; R26R/EYFP* transgenic mouse embryos using fluorescence-activated cell sorting (FACS). Through our characterization, we have defined reproducible benchmarks that track the progression of differentiation *in vitro*, many of which mimic well-documented changes described during these processes *in vivo*. In addition, because we isolated the cranial and trunk NC cells from the same embryos, we were able to directly compare differentiation within the two cell populations. This enabled us to detect, in some cases, subtle differences in cell morphology and behavior as cells differentiated. By establishing a chronological baseline for how the primary cranial and trunk NC cells differentiate under normal conditions, this culture system provides a platform for future investigations assessing how genetic manipulation or exposure to environmental toxins might disrupt the timing of NC differentiation.

## RESULTS AND DISCUSSION

### Cranial and trunk NC cells maintain expression of genes associated with NC cell identity and self-renewal *in vitro* over time

**Fig. 1.**
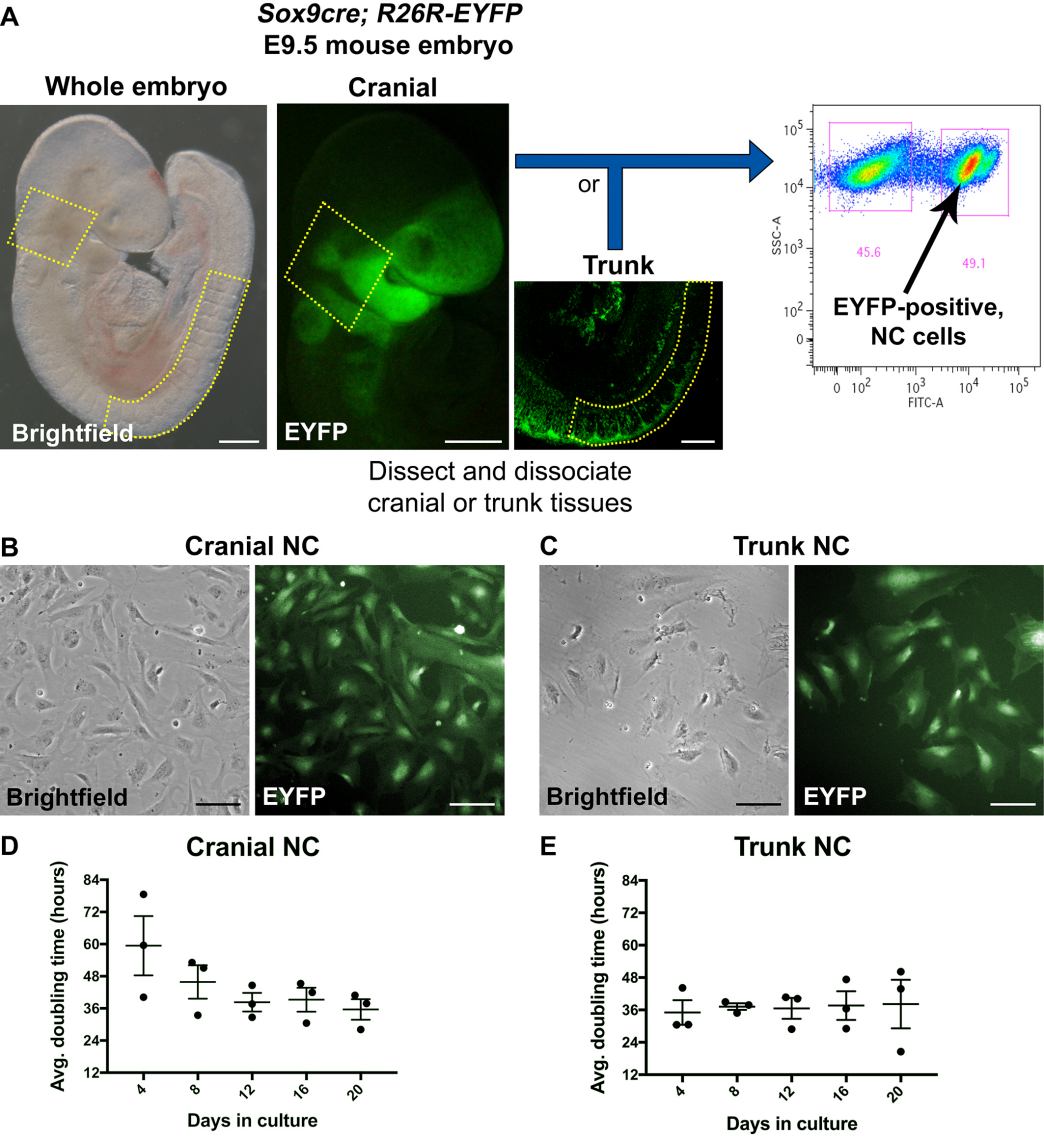
**Isolation and growth profile for primary cranial and trunk NC cells in culture.** (A) Workflow for isolating primary NC cells via FACS. Cranial or trunk tissues were dissected (dotted lines) from *Sox9cre; R26R-EYFP* mouse embryos at E9.5. Tissues were dissociated into single cells and sorted via FACS. Sorted EYFP-positive NC cells were cultured in basal medium and passaged every 4 days. (B) Cranial NC cells. (C) Trunk NC cells. (D,E) Doubling times for cultured cranial (D) and trunk (E) NC cells were calculated over five passages. Average doubling time was 44 h and 39 h for cranial and trunk NC, respectively. Neither cell population showed a statistically significant difference in doubling time across the five passages (repeated measures one-way ANOVA). All cells are derived from *Sox9cre; R26R-EYFP* mice and express EYFP (green). Values represent mean±s.e.m. (*n*=3). Scale bars: (A) 200 µm; (B,C) 100 µm.

Primary cranial and trunk NC cells were separately isolated from embryonic day (E) 9.5 mouse embryos on the basis of *Sox9* reporter gene expression. Cranial tissues were dissected rostral of the otic vesicle, excluding the pharyngeal arches and frontonasal process, and trunk tissues were dissected between somite 8 and somite 24 to avoid isolation of the vagal NC (Fig. 1A, dotted lines). Cranial and trunk tissues isolated from several embryos were separately pooled and dissociated into single cells prior to FACS. Sorted EYFP-positive cells cultured in basal medium exhibited a mesenchymal, stellate cell morphology (Fig. 1B,C), as previously described for NC cells in culture (Ishii et al., 2012; Maurer et al., 2007).

Our method for isolating murine NC cells via FACS differs from previously established protocols, in which NC cells are collected after emigrating from neural tube explants dissected at E8.5. At E8.5, isolating premigratory NC cells via FACS alone does not yield enough cells to establish successful growth in culture. Using Wnt1 as a marker, FACS has been used to further select NC cells isolated from E8.5 neural tube explants and expanded in culture (Ishii et al., 2012). Although Wnt1 is commonly used as a marker for selecting the NC (Druckenbrod and Epstein, 2005; Ishii et al., 2012; Pfaltzgraff et al., 2012; Wong et al., 2006), Wnt1 is expressed throughout the cranial dorsal neural tube, where it is a marker for both NC and non-NC derivatives (Lewis et al., 2013; McMahon and Bradley, 1990; Pietri et al., 2003). This makes it difficult to specifically isolate cranial NC cells directly from the embryo by FACS using Wnt1 as a marker. In contrast, our method isolates *Sox9*-positive NC cells from specific tissues dissected at E9.5, where Sox9 is a well-established marker for both premigratory and migratory NC cells (Cheung and Briscoe, 2003). In addition, by isolating primary NC cells at this later developmental time point, we were able to consistently isolate enough purified NC cells to establish successful cultures without the need for an intermediate *in vitro* expansion step prior to selection (Ishii et al., 2012).

**Fig. 2.**
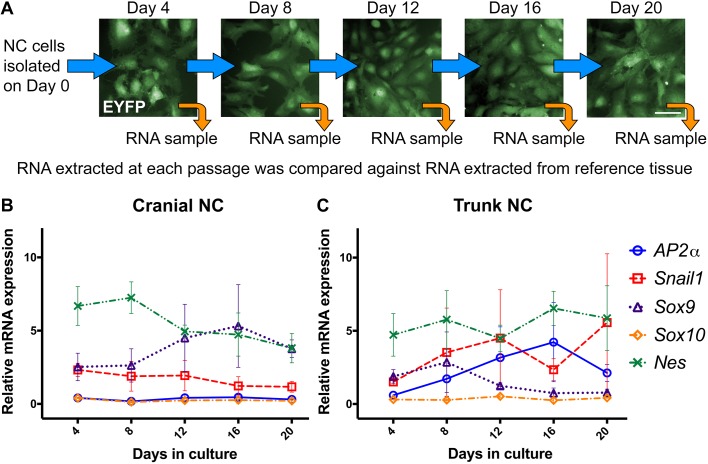
**Maintenance of gene expression associated with NC cell identity and self-renewal.** (A) Workflow for RT-qPCR analysis. RNA was extracted from a sample of cells at each passage. Expression of NC cell markers (*AP2α*, *Snail1*, *Sox9*, *Sox10*), and that of stem cell-like marker (*Nes*), were assessed every 4 days as cells were passaged over 20 days in culture. The cultured NC cells displayed a mesenchymal morphology at each time point across three independent cell isolates. (B,C) Neither the cranial (B) nor the trunk (C) NC cells showed a statistically significant change in the relative expression of these genes across five passages (two-way ANOVA). All cells are derived from *Sox9cre; R26R-EYFP* mice and express EYFP (green). Values represent mean±s.e.m. (*n*=6; three independent cell isolates carried out in duplicate). Scale bar: 50 µm.

Once in culture, both NC cell populations expanded relatively quickly while maintaining their characteristic morphology. Cells were passaged every 4 days over a 20-day period. Although cranial NC cell growth was variable during the first 4 days in culture, the cells maintained consistent growth over subsequent passages (Fig. 1D). The trunk NC cells maintained consistent growth throughout the 20-day period (Fig. 1E). On average, cranial and trunk NC cultures doubled approximately every 40 h and maintained high viability (93±4.49%) based on Trypan Blue exclusion. Overall, the mesenchymal, stellate cell morphology was retained over several passages during the same time period (Fig. 2A), although we sporadically observed isolated cells exhibiting short processes and neuronal morphology. We typically isolated ∼9000 cranial and 3000 trunk NC cells/embryo. Given the average doubling times of the cells, over 7 million cranial and 5 million trunk NC cells can be obtained from an average litter size of nine embryos after just three passages (12 days in culture; Table S1). Thus, *Sox9*-positive NC cells isolated and cultured in this manner display a robust capacity for survival and expansion *in vitro*.

Furthermore, the cultured cells retained their NC cell identity and capacity for self-renewal over time. We used quantitative reverse transcriptase-polymerase chain reaction (RT-qPCR) to assess the relative expression of several well-described markers of NC cell fate, including *AP2α* (also known as *Tfap2a*), *Snail1*, *Sox9* and *Sox10*, and stem cell-like marker *Nes*. Expression analysis was performed using RNA extracted from a sample of the cultured cranial or trunk NC cells every 4 days, as cells were passaged over a 20-day period (Fig. 2A). Using this approach, we found that neither cell population exhibited a statistically significant change in the relative expression of these genes across the various time points (Fig. 2B,C). Together with the growth profile and morphological observations of the cultured cells, these results indicate that NC cell identity and self-renewal capacity is maintained as cells are expanded over a 3-week period.

### Cranial and trunk NC cells differentiate into multiple NC derivatives *in vitro*

**Fig. 3.**
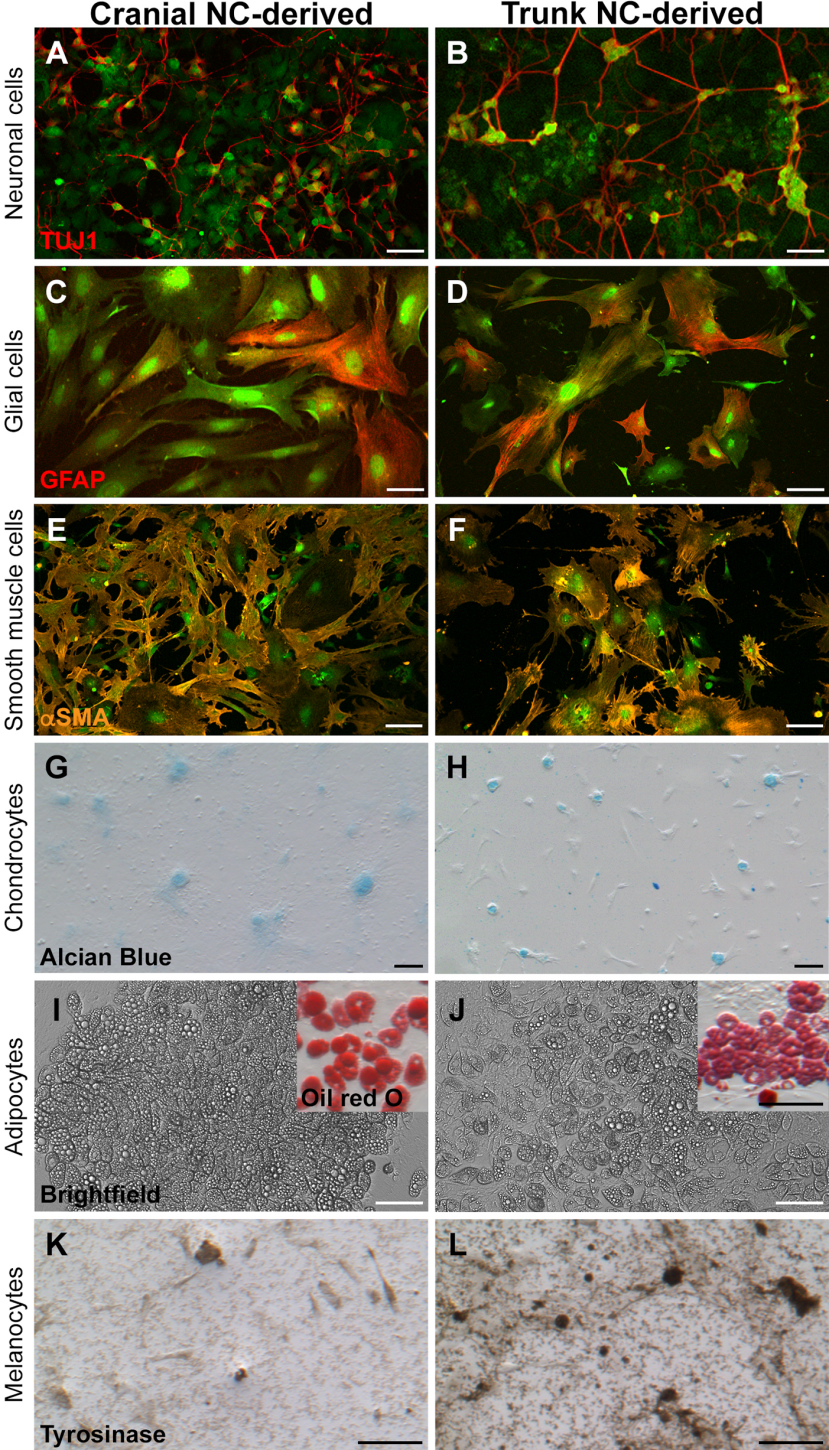
**Differentiation potential of cultured primary cranial and trunk NC cells.** Both cranial and trunk NC cells gave rise to known NC derivatives when grown under conditions reported to induce lineage-specific differentiation. (A–L) Representative images of differentiated cells: neuronal cells (A,B; TUJ1, red; 4 days in differentiation medium containing NT3 and NGF), glial cells (C,D; GFAP, red; 8 days in differentiation medium containing BMP2 and LIF), smooth muscle cells (E,F; αSMA, orange; 7 days in differentiation medium containing FCS), chondrocytes (G,H; Alcian Blue staining; 6 days (G) and 14 days (H) in differentiation medium containing TGF-β3), adipocytes [I,J; brightfield (insets, Oil Red O staining); 14 days in Adipogenic Medium from STEMCELL Technologies] and melanocytes (K,L; tyrosinase, brown; 10 days in differentiation medium containing ET3). All cells are derived from *Sox9cre; R26R-EYFP* mice and express EYFP (green). Cells were expanded for three passages (12 days in culture) prior to differentiation. Differentiation into each of the derivatives was consistent across replicates (*n*=6; duplicate cultures from each of three independent cell isolates). Scale bars: (A–D) 50 µm; (E–L) 100 µm.

A hallmark of NC cells *in vivo* is their ability to give rise to a diverse range of cell types including ectodermal and mesoectodermal derivatives (Le Douarin, 1982). Therefore, we tested the ability of cultured *Sox9*-positive cranial and trunk NC cells to produce various cell types when exposed to lineage-specific differentiation conditions. Our results show that both cell populations could be induced to generate a broad array of NC cell derivatives *in vitro*, including neuronal cells (Fig. 3A,B; Fig. S1), glial cells (Fig. 3C,D), smooth muscle cells (Fig. 3E,F; Fig. S2), chondrocytes (Fig. 3G,H; Fig. S3), adipocytes (Fig. 3I,J; Fig. S3) and melanocytes (Fig. 3K,L; Fig. S3). For consistency, differentiation potential was assessed after passaging the cells three times (12 days in culture); however, we have been successful in inducing differentiation of ectodermal and mesoectodermal derivatives at both earlier and later passages as well (data not shown).

As previously described, the cultured cells can give rise to a larger repertoire of derivatives than would be expected from the same cells *in vivo*, when exposed to appropriate differentiation factors. For example, cultured trunk NC cells grown under conditions that simulate the endogenous microenvironment during differentiation could give rise to chondrocytes (Ido and Ito, 2006; Maurer et al., 2007; McGonnell and Graham, 2002) and adipocytes (Billon et al., 2007), albeit with less efficiency compared with cranial NC cells. To ensure that differentiation into mesoectodermal derivatives was not due to inadvertent expansion of mesodermal cells, we tested for the expression of canonical mesodermal markers, *T* (brachyury) and *Tbx6*. We did not detect expression of either marker in cultured cranial or trunk NC cells (Fig. S4). Thus, at least some NC cells seem to possess an inherent plasticity concerning fate determination that can be influenced by exposure to the appropriate environmental cues.

Although both cranial and trunk NC cells were able to produce all the differentiated cell types tested, we observed that melanocyte differentiation appeared more robust in trunk NC cultures, whereas chondrogenic differentiation appeared more robust in cranial NC cultures. The subtle variations in the extent of differentiation observed between cranial and trunk NC could indicate an intrinsic difference in the way that these two populations respond to environmental cues. Alternatively, these differences might reflect mixtures of progenitors with varying differentiation potential, as has been described previously (Morrison et al., 1999; Stemple and Anderson, 1992). In support of this explanation, recent studies have revealed that a large majority of premigratory/migratory murine trunk NC cells are multipotent, both as single cells and as a population (Baggiolini et al., 2015). However, the number of derivatives formed by each single cell varied, consistent with the premise that NC cells exhibit diverse multipotent potentials. Nevertheless, these data demonstrate that the cultured *Sox9*-positive cranial and trunk NC cells maintain a broad differentiation potential *in vitro*, as would be expected from this multipotent cell population. Expanding on these findings, we next sought to establish reproducible benchmarks exhibited by the primary cranial and trunk NC cells as they differentiated towards the neuronal, glial and chondrogenic cell fate *in vitro* over time.

### Cranial and trunk NC cells exhibit reproducible, temporal changes in morphology and behavior during neuronal differentiation *in vitro*

**Fig. 4.**
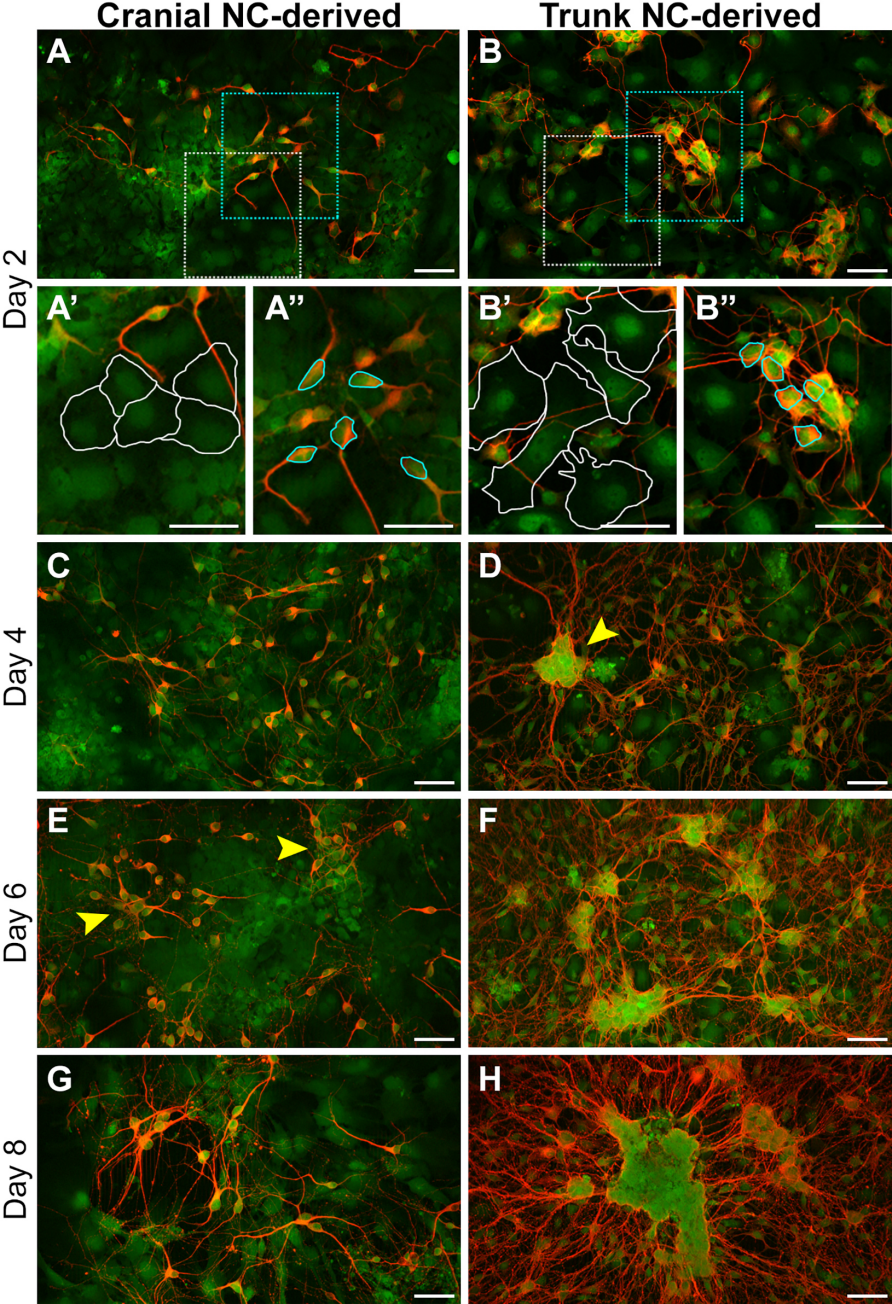
**Temporal progression of neuronal differentiation in cultured cranial and trunk NC cells.** (A–H) Neuronal differentiation was assessed at various time points via immunostaining for TUJ1, a neuron-specific class III β-tubulin. Robust expression of TUJ1 was observed at Day 2 (A,B). Higher magnification views of the boxed regions show an observable decrease in soma size when comparing TUJ1-negative cells displaying a mesenchymal morphology (A′,B′; white lines) and cells positive for TUJ1 that display a neuronal-like morphology (A″,B″; cyan lines). Neuritic outgrowth continued through Day 4 in both cell populations (C,D). In addition, in trunk NC-derived cells, TUJ1-positive cells formed discrete aggregates (D; arrowhead); however, similar aggregation was not observed in cells derived from the cranial NC at this time point. By Day 6, the difference in aggregation between the cell populations became more apparent (E,F). Whereas TUJ1-positive aggregates derived from the trunk NC were tightly compacted (F), TUJ1-positive aggregates derived from the cranial NC were loosely formed (E; arrowheads). At Day 8, TUJ1-positive cells derived from the cranial NC maintained a similar phenotype as seen in Day 6 (G). In contrast, TUJ1-positive cells derived from the trunk NC displayed enhanced aggregation, coupled with extensive neuritic outgrowth (H). Phenotypic characteristics of the cells at each time point were consistently observed (*n*=6; duplicate cultures from each of three independent cell isolates). All cells are derived from *Sox9cre; R26R-EYFP* mice and express EYFP (green). Red staining=TUJ1. Scale bars: 50 µm.

**Fig. 5.**
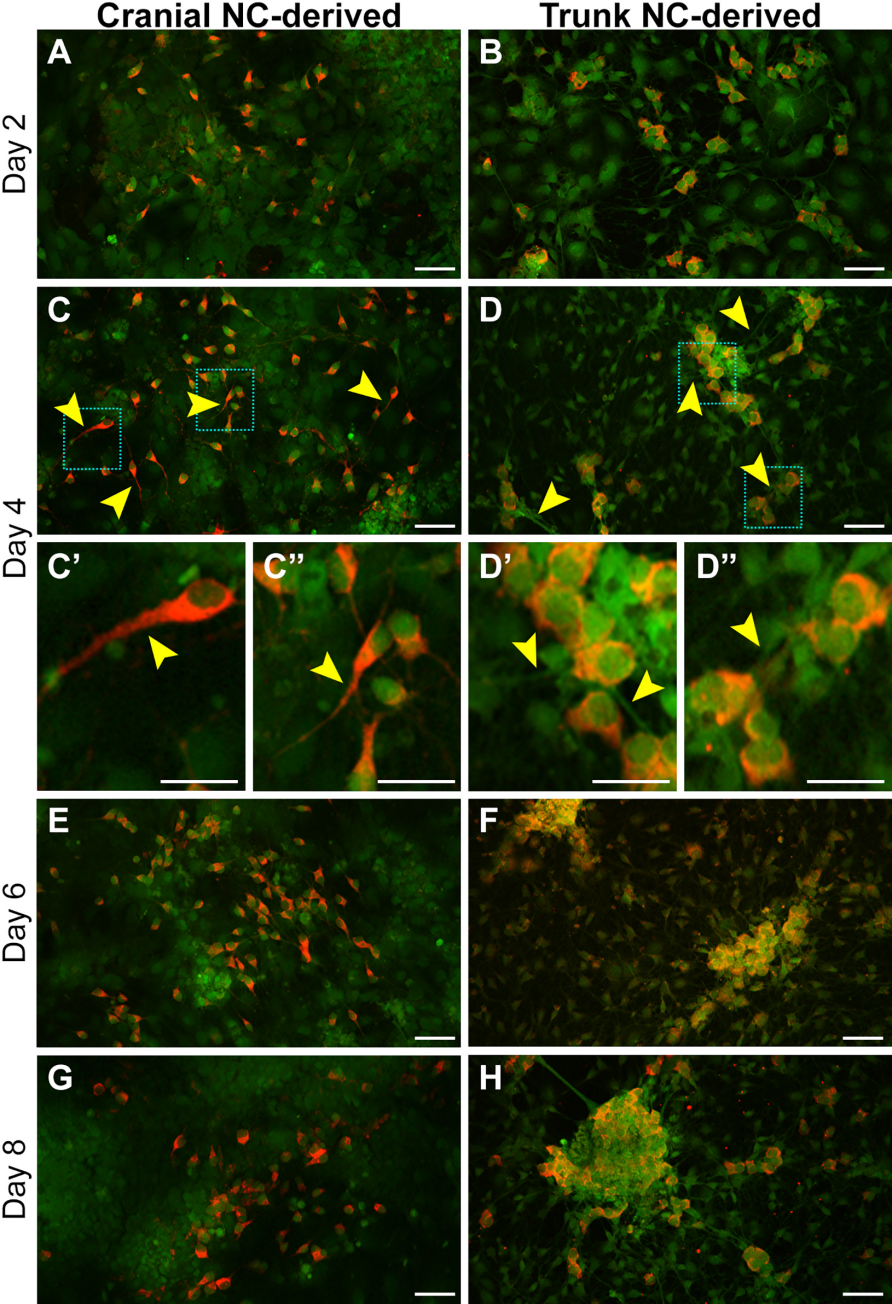
**Cultured cranial and trunk NC cells exhibit differential HuC/D localization during neuronal differentiation.** Neuronal differentiation was assessed at various time points via immunostaining for HuC/D, a pan-neuronal marker recognizing neuron-specific RNA-binding proteins. Robust HuC/D expression was observed after 2 days in differentiation medium (A,B). By Day 4, a difference in HuC/D localization in neuritic processes between the cell populations was observed (C,D; arrowheads). Higher magnification views of the boxed regions show HuC/D localized to the soma and neuritic process of cells derived from the cranial NC (C′,C″; arrowheads); however, HuC/D expression was only observed in the soma of cells derived from the trunk NC and was absent from the neuritic processes (D′,D″; arrowheads). Differences in HuC/D localization between the cell populations persisted through Day 8 (E–H). Phenotypic characteristics of the cells at each time point were consistently observed (*n*=6; duplicate cultures from each of three independent cell isolates). All cells are derived from *Sox9cre; R26R-EYFP* mice and express EYFP (green). Red staining=HuC/D. Scale bars: (A–D,E–H) 50 µm; (C′,C″,D′,D″) 25 µm.

Neuronal differentiation was characterized by assessing the expression of neuron-specific class III β-tubulin (TUJ1; also known as Tubb3) and ELAV-like RNA binding proteins (HuC/D; also known as Elavl3/Elavl4) at several time points along the 8-day differentiation process (Figs 4 and 5). Robust expression of both markers was observed as early as Day 2 in neuronal differentiation medium (Fig. 4A,B; Fig. 5A,B). In addition, there was an observable decrease in soma size in differentiated cells, as illustrated in cultures immunostained with TUJ1 (Fig. 4A,B). Specifically, TUJ1-negative cells displayed a stellate, mesenchymal morphology (Fig. 4A′,B′, white lines), similar to the morphology seen in undifferentiated cells shown previously (Fig. 1B,C). In contrast, TUJ1-positive cells exhibited neuronal-like morphology with a compacted soma (Fig. 4A″,B″, cyan lines), as well as the extension of neuritic processes.

Neuritic outgrowth continued in both cell populations as differentiation progressed through Day 4 (Fig. 4C,D) and Day 6 (Fig. 4E,F); however, under these culture conditions, we observed clear differences in cell aggregation. After 4 days in differentiation medium, TUJ1-positive cells derived from the trunk NC began to coalesce in distinct regions, forming dense aggregations (Fig. 4D; Fig. S1, arrowheads). Similar coalescence was not observed in TUJ1-positive cells derived from the cranial NC until Day 6. In addition, the aggregates formed by cranial NC were more loosely formed and amorphous (Fig. 4E, arrowheads) compared with those observed in cultures of trunk NC. Aggregates observed at Day 6 in cranial NC resembled those observed at Day 2 in cells derived from the trunk NC (Fig. 4B, cyan dotted line box). The loose aggregation of TUJ1-positive cells and moderate neuritic outgrowth, remained characteristic of the cranial NC-derived cells as differentiation progressed through Day 8 (Fig. 4G; Fig. S1). This was in stark contrast to the aggregates formed from trunk NC-derived positive cells, which were more compact and well defined at Day 6 (Figs 4F and 5F) and Day 8 (Figs 4H and 5H; Fig. S1), and displayed extensive neuritic outgrowth. The changes in morphology and timing of aggregation were consistently observed over multiple replicates (*n*=8) from several different cell isolates (*n*=4). Therefore, these characteristics can all be used to assess the temporal progression of neuronal differentiation *in vitro*.

Notably, the temporal progression of neuronal differentiation we observed *in vitro* mimics distinct attributes of this process *in vivo*. During either cranial or dorsal root ganglion assembly *in vivo*, migrating NC cells are directed into discrete streams, which promote aggregation and subsequent neuronal differentiation. Although we observed aggregation in both cell populations, the trunk NC-derived neurons displayed a greater propensity for aggregation (Fig. 4; Fig. S1). *In vivo*, aggregation is a common behavior observed between the cranial and trunk NC during gangliogenesis; however, there are inherent differences in cell-environment interactions during this process. Whereas the dorsal root ganglia arise exclusively from the trunk NC, neurons in the cranial ganglia are largely derived from placodal ectoderm in the head, with contributions from the NC. To date, the specific signals involved in regulating interactions between NC and placodal ectoderm involved in cranial ganglion assembly are still under investigation (Kurosaka et al., 2015). Based on the differences we observe between the cranial and trunk NC-derived neurons, this *in vitro* culture system provides an excellent tool for investigating potential factors that promote aggregation during gangliogenesis *in vivo*.

Another difference detected in neurons derived from trunk NC was the localization of the ELAV-like RNA binding proteins HuC and HuD. After 4 days in differentiation medium, HuC/D was localized to the soma and neuritic processes in most of the cranial NC-derived cells (85.89±13.12%; Fig. 5C–C″, arrowheads; quantified in Fig. S5). In comparison, HuC/D expression was observed in the soma in trunk NC-derived cells, and mostly absent from the neuritic processes (8.51±12.91%; Fig. 5D–D″, arrowheads; quantified in Fig. S5). Differential HuC/D localization persisted through Day 8 (Fig. 5E–H).

This axial difference suggests that HuC/D is not necessary for neuritic outgrowth during trunk NC-derived neuronal differentiation. HuC and HuD proteins bind to adenylate-uridylate-rich regulatory sequences in the 3′ UTR of target gene transcripts, leading to mRNA stability (Deschênes-Furry et al., 2006). Overexpression of HuC or HuD accelerates neurite outgrowth in cultured PC12 cells (Akamatsu et al., 1999; Anderson et al., 2000), E19 rat cortical neurons and retinoic acid-induced embryonic stem cells (Anderson et al., 2001), and stabilizes growth-associated mRNAs localized to the growth cones of PC12 cells (Smith et al., 2004). Given that we observe HuC/D in neurites of neurons derived from cranial NC, but not in those of neurons derived from trunk NC, it is possible that another type of RNA-binding protein stabilizes growth-associated mRNAs in the trunk NC. Having the ability to detect such differences allows further investigation to determine the specific role of HuC/D, and that of other RNA binding proteins, during neuritogenesis in the peripheral nervous system.

### Cranial and trunk NC-derived cells differentiated in culture display distinct morphological transitions characteristic of glial differentiation *in vivo*

**Fig. 6.**
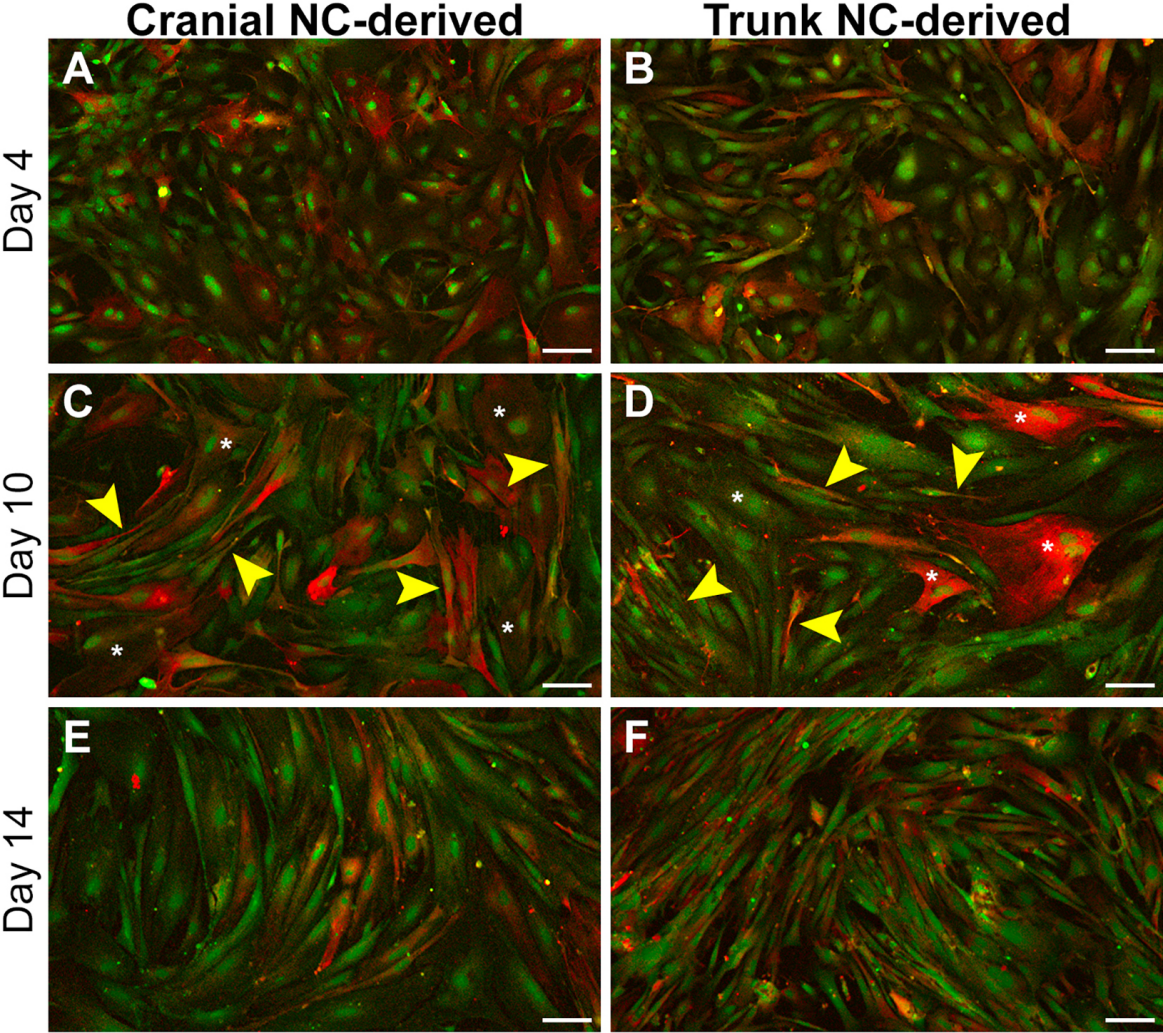
**Primary cranial and trunk NC cells display distinct morphological transitions during the temporal progression of glial differentiation *in vitro*.** Glial differentiation was assessed via immunostaining for GFAP after 4, 10 or 14 days in differentiation medium. GFAP-positive cells were observed in both cranial and trunk NC cell populations at Day 4 (A,B). By Day 10, some of the GFAP-positive cells extended flattened, sheet-like processes (C,D; asterisks) while the other GFAP-positive cells displayed an elongated, spindle-like morphology (C,D; arrowheads). After 14 days, most GFAP-positive cells in both populations exhibited an elongated, bipolar morphology and cells extending flattened, sheet-like processes were only occasionally observed (E,F). Phenotypic characteristics of the cells at each time point were consistently observed (*n*=6; duplicate cultures from each of three independent cell isolates). All cells are derived from *Sox9cre; R26R-EYFP* mice and express EYFP (green). Red staining=GFAP. Scale bars: 100 µm.

The temporal progression underlying glial differentiation was determined utilizing the expression of glial fibrillary acidic protein (GFAP; Fig. 6; Jessen and Mirsky, 1984). After 4 days, robust GFAP expression could be seen in both cranial and trunk NC cells (Fig. 6A,B), with most cells displaying a stellate, mesenchymal morphology comparable to that of undifferentiated cells shown previously (Fig. 1B,C). After 10 days in glial differentiation medium, the cells exhibited a more diversified range of cell morphologies. Specifically, some of the GFAP-positive cells displayed flattened, sheet-like processes (Fig. 6C,D, asterisks), similar to those formed by Schwann cell precursors, while other cells had adopted an elongated, spindle-like morphology (Fig. 6C,D, arrowheads), indicative of immature Schwann cells. By Day 14, most GFAP-positive cells derived from either cell population displayed an elongated morphology, with extension of bipolar processes (Fig. 6E,F). Moreover, flattened, sheet-like processes noted at earlier time points were only observed sporadically throughout the cultures at Day 14. Similar observations were also made using a marker of Schwann cell differentiation, Erb-B2 receptor tyrosine kinase 3 (Erb-B3; Riethmacher et al., 1997; Fig. S6).

The transition from flattened, sheet-like processes to elongation of bipolar processes is considered a hallmark of differentiation during Schwann cell development (Dong et al., 1999; Jessen et al., 2015; Maurer et al., 2007; Thaxton et al., 2011). Changes in cell morphology over the 14-day differentiation time course were highly consistent between cultures (*n*=6) derived from different cell isolates (*n*=3). Therefore, the temporal transition in cell shape, specifically between 10 and 14 days in differentiation medium, could act as a reproducible gauge of differentiation towards the glial cell fate *in vitro*. *In vivo*, further maturation is dependent on environmental context as immature Schwann cells will either become a myelinating or non-myelinating Schwann cell, depending on the size of the associated axon. In the absence of peripheral axons, we conclude that the primary cranial and trunk NC cells are able to give rise to immature Schwann cells *in vitro*, and exhibit a similar capacity to form this cell type. We further conclude that both the primary cranial and trunk NC cells exhibit a similar capacity to differentiate into glial cells, and that differentiation occurs along a similar temporal progression.

### Cranial NC-derived chondrocytes form distinct nodules and secrete measurable cartilage matrix proteins when cultured as a micromass

Cranial NC-derived chondrogenic differentiation was assessed by documenting changes in protein expression and cell morphology at several time points over a 2-week differentiation window. These characteristics were determined utilizing expression of Type II collagen (Col2a1), an established marker of articular chondrocyte differentiation (Lefebvre et al., 1997), as well as staining with Alcian Blue, a common dye used to stain the acidic glycosaminoglycans and sulfated glycoproteins secreted by chondrocytes during chondrogenesis (Mello and Tuan, 1999; Paulsen et al., 1988; Paulsen and Solursh, 1988). *In vivo*, chondroprogenitors initially exhibit a stellate, mesenchymal morphology and subsequently transition to a cuboidal shape as differentiation progresses (Woods et al., 2007). In addition, they begin to produce cartilage matrix proteins, including Col2a1, and highly sulfated proteoglycans, such as aggrecan (Woods et al., 2007). The production of cartilage matrix proteins promotes aggregation, which results in the formation of chondrogenic condensations, a hallmark of overt differentiation (Hall and Miyake, 2000). *In vitro*, we demonstrate that our cultured cells undergo similar transitions in cell morphology, condensation and cartilage matrix secretion as described for chondrogenic progenitors *in vivo.*

**Fig. 7.**
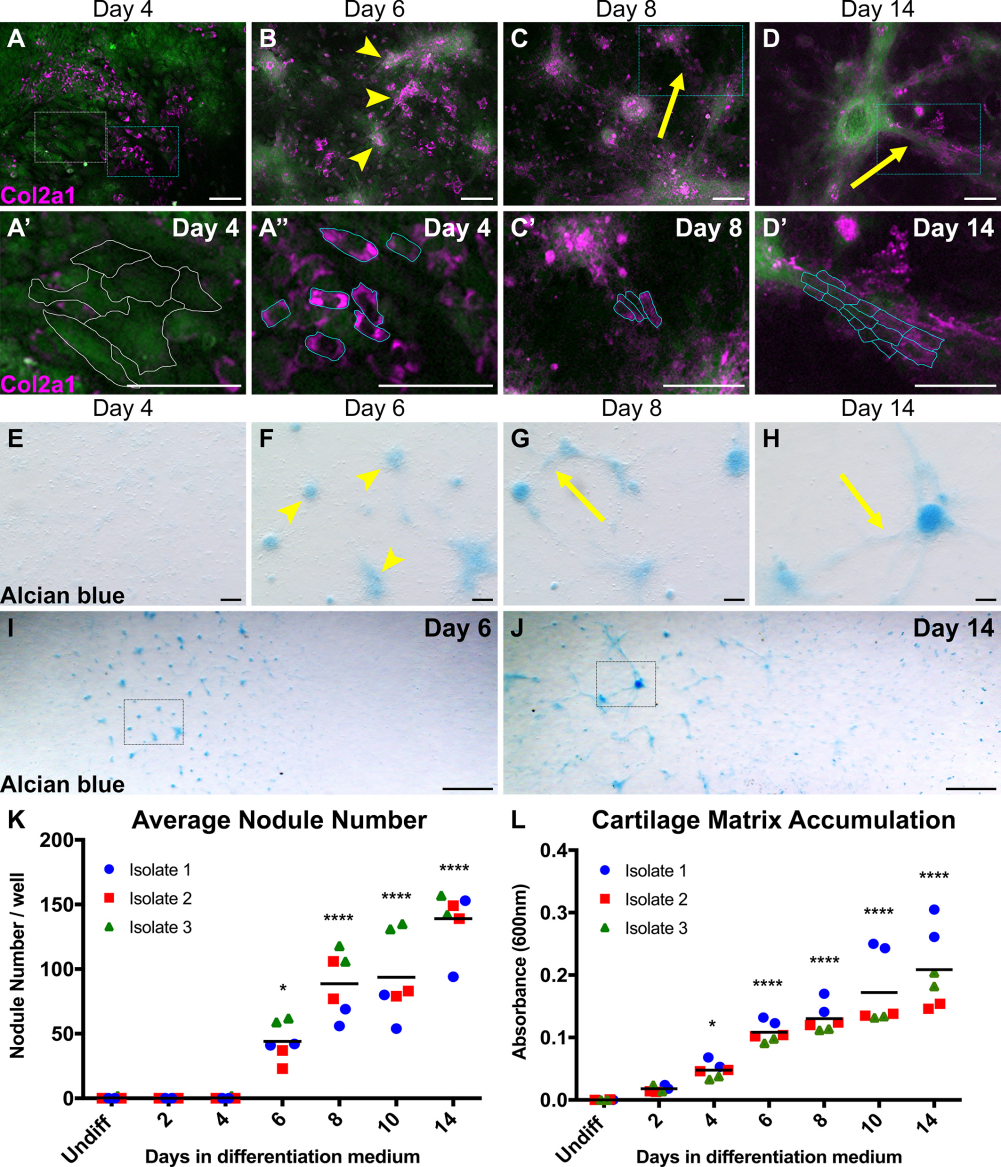
**Cranial NC-derived chondrocytes form distinct nodules and produce cartilage matrix *in vitro* over time.** (A–J) Cell morphology and cartilage matrix production were analyzed at various time points via immunostaining for Type II collagen (Col2a1) (A–D), and Alcian Blue staining (E–J). After 4 days in differentiation medium, Col2a1-positive cells displayed a cuboidal morphology (A). Higher magnification views of the boxed regions highlight the difference between Col2a1-negative cells, which display a mesenchymal morphology (A′; white lines), and Col2a1-positive cells, which display a cuboidal morphology (A″; cyan lines). At this same time point, parallel cultures showed faint Alcian Blue staining (E). By Day 6, Col2a1-positive cells began to form chondrogenic nodules that stained positive for Alcian Blue (B,F; arrowheads). At later stages of chondrogenic differentiation, chains of chondrocytes producing cartilage matrix could be seen emanating from the nodules (C,D,G,H; arrows). Higher magnification views of the boxed regions show chondrocytes that appear aligned, forming chains of cells that emanate from the nodules at Day 8 and Day 14 (C′,D′; cyan lines). In addition, the number of chondrogenic nodules increased over time. (I,J) In order to obtain a representative field of view of the entire culturing surface, four images from overlapping fields of view were aligned and stitched using the open source Hugin software (http://www.hugin.sourceforge.net). Nodules were first observed in distinct regions of the well at Day 6 (I), but by Day 14 had spread throughout the well (J). Boxed regions in I and J correspond to the higher magnification images in F and H, respectively. (K,L) Quantification of chondrogenic nodule number (K) and cartilage matrix accumulation (L) further demonstrates the increase in nodule formation over time. Experiments were repeated from three independent cell isolates, each in duplicate. Each dot represents one technical replicate, grouped by biological isolate. Black horizontal lines indicate the grand mean across replicates. *P<0.05, ****P<0.0001 vs undifferentiated cells (two-way ANOVA). All cells are derived from *Sox9cre; R26R-EYFP* mice and express EYFP (green). Magenta staining=Col2a1. Scale bars: (A–H) 100 µm; (I,J) 1 mm.

After 4 days in differentiation medium, many of the cultured cells positive for Col2a1 had transitioned from a mesenchymal morphology (Fig. 7A,A′, white lines) to a cuboidal one (Fig. 7A,A″, cyan lines). However, at this time point, parallel cultures displayed only faint Alcian Blue staining (Fig. 7E). This suggests that early Col2a1 expression could be promoting the morphological changes necessary to begin the differentiation process, but the robust secretion of cartilage matrix proteins has not yet occurred.

We first observed distinct chondrogenic nodules after 6 days in differentiation medium (Fig. 7B,F, arrowheads). Nodules could be visualized using both Col2a1 and Alcian Blue staining, indicating that cartilage matrix proteins were being produced and secreted. Furthermore, the timing of nodule formation was consistent across multiple replicates (*n*=6) from several distinct cell isolates (*n*=3).

By Day 8, cartilage matrix-producing chondrocytes were observed extending from the nodules (Fig. 7C,G, arrows), a phenotypic characteristic that became more prominent as differentiation progressed through Day 14 (Fig. 7D,H, arrows). Higher magnification views of the boxed regions in Fig. 7C and D show chondrocytes, which appeared to align and form chains of cells that emanate from the nodules at Day 8 (Fig. 7C′, cyan lines) and Day 14 (Fig. 7D′, cyan lines).

Based on our observations, increases in nodule number and cartilage matrix accumulation over time can serve as a measurable benchmark of chondrogenic differentiation *in vitro*. Lower magnification views at Day 6 revealed that chondrogenic nodule formation occurred in localized regions of the cultures (Fig. 7I). However, by Day 14, nodules were observed throughout the cultures (Fig. 7J). Further quantification of temporal changes in nodule number and cartilage matrix accumulation were analyzed in Alcian Blue-stained cultures. Consistent with our histological observations, our results showed a statistically significant increase in both nodule number (Fig. 7K) and cartilage matrix accumulation (Fig. 7L) over time when compared with the undifferentiated cells. Together, these data reveal several reproducible characteristics that define the temporal progression of cranial NC-derived chondrogenic differentiation *in vitro*, including the timing of detectable Alcian Blue staining, nodule formation and secretion of cartilage matrix proteins.

### Significance and impact

Distinguishing how cell intrinsic factors and cell environment contribute to the broad, but varied, differentiation potential of NC cells is critical to understanding embryonic development and NC-related diseases. Gene regulatory networks governing NC specification, migration and differentiation demonstrate that NC-specifier genes can play multiple roles over the course of NC development (Martik and Bronner, 2017). For example, Sox9 plays important roles in premigratory and migratory NC, and is also a key regulator of chondrogenic differentiation in cranial NC. Although we demonstrate broad potential for differentiation of Sox9-positive NC cells, it is possible that our primary cultures select for a population of cells that have a special propensity for Sox9-dependent differentiated cell types. Alternatively, it is possible that Sox9, which is co-expressed broadly in migrating NC cells with other NC specifiers, is later re-expressed in a more limited population during differentiation. We anticipate that the isolation and expansion methods we have described could also be applied to embryos from the broad array of NC reporter lines (Debbache et al., 2018). Genomic and proteomic analyses of NC cells isolated based on the expression of different NC specifiers could help to determine whether expression of different specifiers select for distinct subpopulations with variations in differentiation potential.

Our comparison of cranial and trunk NC isolated from the same animals has also enabled us to detect axial differences in neuronal and chondrogenic differentiation. The most significant axial differences we noted was in the ability of cells to aggregate during neuronal and chondrogenic differentiation. Future studies may exploit these differences by using genomic and proteomic analyses to identify cell intrinsic factors that contribute to gangliogenesis or chondrogenic condensation. Another difference we detected was in the cellular localization of the HuC/D RNA-binding proteins in neurons derived from cranial or trunk NC. This suggests that axonal trafficking, or local translation within peripheral axons arising from neurons in the head and trunk, may be governed by different mechanisms. Our culture model provides a means for isolating neurites from the different populations to address this possibility. In addition, these cultures could be used to screen pharmacological agents that promote or disrupt these same processes.

A unique contribution of the current study is our temporal characterization of differentiation along the neuronal, glial and chondrogenic lineages. Through this analysis, we reproducibly identified distinct intermediate stages during differentiation that mimicked those previously described *in vivo*. We envision that future studies will elucidate gene regulatory changes that accompany these transitions, which could identify cell intrinsic drivers of stage-specific progression along differentiation pathways. Such drivers would serve as important targets for screening potential therapeutic agents useful in stem cell therapies; for example, agents that promote aggregation of cells during chondrogenic differentiation. In addition, our temporal characterization of differentiation *in vitro* provides a basis for more comprehensive testing of industrial compounds, personal use products and therapeutic drugs for developmental toxicity. Although whole-animal testing remains the standard for assessing developmental toxicity, the cost and time involved in *in vivo* testing has resulted in a large backlog of compounds to be tested (Woodruff et al., 2011; Mitro et al., 2015). *In vitro* assays that include intermediate endpoints in neuronal, glial and chondrogenic differentiation could serve as a valuable preliminary screen to prioritize compounds for further *in vivo* analysis.

Another potential application of this culture system is to examine cell-intrinsic mechanisms of NC differentiation in the context of various mouse models of human NC-related disorders. Disruption of NC development underlies many pediatric syndromes and has been associated with deletions in multiple genes affecting various aspects of NC development. In many neurocristopathies, patients with identical deletions present with widely disparate symptoms or with varying severity of symptoms (Noack Watt and Trainor, 2014). This suggests a critical interplay between genetic and environmental factors that can contribute to the severity of NC-related disorders. *In vitro* methods, such as ours, enable the characterization of cell-intrinsic contributions of specific mutations, which can subsequently be assessed in the context of controlled environmental manipulations. For example, the *LgDel* mouse model of chromosome 22q11 deletion syndrome displays many of the hallmarks of the pediatric syndrome including dysphagia (Karpinski et al., 2014). These mice display defects in both cranial nerve development and orofacial morphology, suggesting an impact of the deletion on neuronal and chondrogenic development. However, *in vivo*, it is difficult to separate the direct effects of the deletion on each tissue from the potential of the neural defect to secondarily impact orofacial development. Furthermore, the ability to partially rescue the phenotype by manipulation of retinoic acid synthesis suggests the potential for environmental disrupters to further modify the effects of the deletion. *In vitro* analysis of differentiation along both the neural and chondrogenic lineages could help to distinguish the direct impact of the deletions on differentiation of each lineage separately, while providing a platform to investigate environmental contributions.

In accordance with our primary objectives, we have developed methods for effective isolation, expansion and directed differentiation of murine NC cells. With this method, we are able to reliably obtain enough cells for biochemical analyses, such as co-immunoprecipitation and ChIP-seq, after only a short expansion in culture, thus advancing our ability to elucidate novel protein-protein and protein-gene interactions that regulate NC growth and differentiation. Additionally, because we isolate both cranial and trunk NC cells from the same embryos, this culture system offers a foundation to tease apart the intrinsic and extrinsic factors that contribute to axial-specific cell fate acquisition. Finally, we established reproducible benchmarks that define the temporal progression of neural and chondrogenic differentiation *in vitro.* These benchmarks significantly enhance our ability to determine how subtle alterations in the timing of differentiation due to genetic mutation or toxicological exposures contribute to NC-related birth defects and disorders.

## MATERIALS AND METHODS

### Experimental animals

All animal procedures were conducted according to the National Institutes of Health Guide for the Care and Use of Laboratory Animals and were approved by the Institutional Animal Care and Use Committee at the University of Wisconsin-Milwaukee. *Sox9-*cre mice, in which an IRES-Cre-pA cassette was inserted within the 3′UTR of the endogenous *Sox9* gene, were a kind gift from Dr Benoit de Crombrugghe (Akiyama et al., 2005)*. B6.129X1-Gt(ROSA)26Sor^tm1(EYFP)Cos^/J* mice (Srinivas et al., 2001), henceforth designated as *R26R-EYFP*, were obtained from The Jackson Laboratory (Bar Harbor, ME, USA). Timed matings between homozygous mice from each strain were conducted to produce *Sox9cre; R26R-EYFP* embryos. The presence of a vaginal plug on the following morning was counted as E0.5.

### Neural crest cell culture

Primary cranial and trunk NC cells were cultured using protocols adapted and modified from Stemple and Anderson (1992), Bixby et al. (2002) and Ishii et al. (2012).

#### Basal medium

The basal medium was prepared as follows (Bixby et al., 2002): Dulbecco's modified Eagle medium (DMEM)-low glucose, pyruvate (Gibco), 30% Neurobasal-A Medium (Gibco), 15% chick embryo extract (prepared as described in Stemple and Anderson, 1992), 55 mM 2-mercaptoethanol (Gibco), 1% N2 supplement (Gibco), 2% B27 supplement (Gibco), 100 U/ml penicillin and 100 µg/ml streptomycin (Corning). Basal medium was sterile filtered (0.22 µm pore size) and supplemented with 25 ng/ml recombinant human basic fibroblast growth factor (bFGF; R&D Systems), 20 ng/ml recombinant human insulin-like growth factor 1 (IGF1; Gibco) and 35 ng/ml retinoic acid (Sigma-Aldrich).

#### Isolation and expansion

Cranial or trunk tissues were specifically dissected from several *Sox9cre; R26R-EYFP* mouse embryos at E9.5 (Fig. 1A). Cranial or trunk tissues were pooled and each pool of tissue was enzymatically dissociated in Accumax (STEMCELL Technologies) prior to FACS (BD FACSAria III flow cytometer, BD Biosciences). Live EYFP-positive cranial or trunk NC cells were grown in basal medium at 37°C, 5% CO_2_ on tissue culture-treated plates/flasks coated with 50 µg/ml poly-D-lysine (Sigma-Aldrich) and 150 µg/ml fibronectin (Akron Biotechnology). Cells were seeded at 30,000 cells/cm^2^ and passaged every 4 days. Approximately half of the basal medium was exchanged every other day. Primary cells isolated via FACS were defined as passage 0 (P0), and each cycle of trypsinization and re-seeding was considered to be an additional passage.

For subculturing, cells were rinsed with phosphate-buffered saline (PBS) and treated with 0.25% trypsin-EDTA (Gibco) at 37°C for 3 min. Cells were neutralized in 10% heat inactivated fetal calf serum (HI-FCS; Gemini Bio-Products) in DMEM and counted prior to re-seeding onto freshly coated plates/flasks. Cultures were typically diluted two to three times, with a target seeding density of 30,000 cells/cm^2^. Under these conditions, the primary cranial and trunk NC cells maintained logarithmic growth for an extended period of time.

#### Quantification of doubling time

Doubling time was determined as cells were passaged every 4 days (96 h) over the course of a 20-day period. At each passage, cells were initially seeded at a density of 30,000 cells/cm^2^, then after 4 days, cells were detached and final live-cell counts were determined using the Trypan Blue exclusion method. Initial and final cell concentrations at each passage were calculated by normalizing against surface area. Doubling time at each passage was determined using an online doubling time calculator (http://www.doubling-time.com/compute.php).

### Gene expression analysis by RT-qPCR

RT-qPCR was performed to assess the relative expression of several markers of NC cell fate (*AP2α*, *Snail1*, *Sox9*, *Sox10*) and stem cell-like properties (*Nes*) as the primary NC cells were passaged over time. *GAPDH* was used as a housekeeping gene. Primers were both designed and checked for specificity using Primer-BLAST (NCBI), then empirically validated prior to use. The following primer pairs were used: *AP2α* (5′-CACTCCTTACCTCACGCCAT-3′, 5′-GCCACCGTGACCTTGTACTT-3′); *Snail1* (5′-CTGCACGACCTGTGGAAAG-3′, 5′-GCCTGGCACTGGTATCTCTT-3′); *Sox9* (5′-AGTCGGTGAAGAACGGACAA-3′, 5′-CCCTCTCGCTTCAGATCAACT-3′); *Sox10* (5′-TTCAGGCTCACTACAAGAGTGC-3′, 5′-ATTACCTCGTGGCTGATCTCC-3′); *Nes* (5′-AACAGAGATTGGAAGGCCGC-3′, 5′-GCCACTTCCAGACTAAGGGAC-3′); *GAPDH* (5′-GCTCATGACCACAGTCCATGC-3′, 5′-GTTGGGATAGGGCCTCTCTTG-3′).

Total RNA was extracted from a sample of the cultured cranial or trunk NC cells every 4 days, as cells were passaged over a 20-day period using the RNeasy Micro kit (Qiagen). After DNase treatment, 250 ng RNA from each sample was transcribed to complementary DNA (cDNA) using the qScript Flex cDNA Synthesis kit (Quantabio) and a mixed primer strategy (Oligo dT_20_ and Random Primer), following the manufacturer’s protocol. cDNA was diluted 1:3 or 1:2 for cranial or trunk NC samples, respectively, prior to being used as a template in a 20 µl reaction containing 10 µl Fast SYBR Green Master Mix (Applied Biosciences) and 125 nmol forward and reverse primers. All RT-qPCR reactions were performed in duplicate on a CFX96 Touch Real-Time PCR Detection System (Bio-Rad). Reactions were amplified using a two-stage protocol: 95°C for 5 min, followed by 44 cycles of 95°C for 15 s, and 60°C for 1 min. After the last cycle, a melt curve was generated. Fold change in gene expression relative to the reference tissue (*AP2α*, *Snail1*, *Sox9*, *Nes*: whole/unsorted E9.5 mouse embryo; *Sox10*: adult murine brain) was calculated for each primer set using the [2^−ΔΔCt^] method (Livak and Schmittgen, 2001).

### Differentiation of NC cultures

For all derivatives, cultured cells were expanded for three passages prior to differentiation. Each differentiation experiment was carried out in duplicate and repeated using three independent cell isolates, for a total of six replicates. In many cases, additional replicates were carried out for experiments described in the Supplementary Information.

#### Generation of neuronal cells

Primary cranial or trunk NC cells were seeded at 10,000 cells/cm^2^ onto tissue culture-treated glass slides coated with 50 µg/ml poly-D-lysine (Sigma-Aldrich) and 10 µg/ml laminin (Corning). Cells were grown in neuronal differentiation medium: Neurobasal-A Medium (Gibco), 2 mM GlutaMAX (Gibco), SM1 neuronal supplement (STEMCELL Technologies), 100 U/ml penicillin and 100 µg/ml streptomycin (Corning). The medium was sterile filtered, then supplemented with 100 ng/ml mouse nerve growth factor 2.5S (NGF; MilliporeSigma) and 50 ng/ml recombinant human neurotrophin-3 (NT3; MilliporeSigma). Half the medium was exchanged every other day.

#### Generation of glial cells

Primary cranial or trunk NC cells were seeded at 12,000 cells/cm^2^ onto tissue culture-treated glass slides coated with 50 µg/ml poly-D-lysine (Sigma-Aldrich) and 10 µg/ml laminin (Sigma-Aldrich). Cells were grown in glial differentiation medium, as previously described (Ishii et al., 2012): DMEM/F12 (Gibco), 1% HI-FCS (Gemini Bio-Products), 2 mM GlutaMAX (Gibco), 2% B27 supplement (Gibco), 100 U/ml penicillin and 100 µg/ml streptomycin (Corning). Medium was sterile filtered, then supplemented with 50 ng/ml recombinant human bone morphogenetic protein 2 (BMP2; Gibco) and 50 ng/ml recombinant human leukemia inhibitory factor (LIF; MilliporeSigma). Half the medium was exchanged every other day.

#### Generation of chondrocytes

Chondrogenic differentiation was induced using methods adapted from those previously described (Ishii et al., 2012). Primary cranial or trunk NC cells were initially cultured as a monolayer (30,000 cells/cm^2^) on tissue culture-treated plates coated with 50 µg/ml poly-D-lysine (Sigma-Aldrich) and 150 µg/ml fibronectin (Akron Biotechnology). Cells were grown for 3 days in osteogenic differentiation medium: α-minimum essential medium (α-MEM; Corning), 10% HI-FCS (Gemini Bio-Products), 0.1 µM dexamethasone (Sigma-Aldrich), 10 mM β-glycerophosphate (Sigma-Aldrich), 50 µg/ml L-ascorbic acid (Sigma-Aldrich), 100 U/ml penicillin and 100 µg/ml streptomycin (Corning). Medium was sterile filtered, then supplemented with 100 ng/ml recombinant human BMP2 (Gibco). Then, on Day 4, cells were harvested and cultured as a micromass (Zhang et al., 2010). Briefly, cells were resuspended at 2×10^7^ cells/ml in a 10 µl droplet. One droplet was placed in the center of each well in a four-well plate. Cells were allowed to adhere at 37°C for 1 h, prior to the addition of 400 µl chondrogenic differentiation medium (Ishii et al., 2012): α-MEM (Corning), 5% HI-FCS (Gemini Bio-Products), 0.1 µM dexamethasone (Sigma-Aldrich), 50 µg/ml L-ascorbic acid (Sigma-Aldrich), 1% ITS+ Premix (Corning), 1 mM sodium pyruvate (Gibco), 100 U/ml penicillin and 100 µg/ml streptomycin (Corning). Medium was sterile filtered, then supplemented with 10 ng/ml recombinant human BMP2 (Gibco) and 10 ng/ml human recombinant transforming growth factor-beta 3 (TGF-β3; Invitrogen). Half the medium was exchanged every other day.

#### Generation of smooth muscle cells

Primary cranial or trunk NC cells were seeded at 30,000 cells/cm^2^ onto tissue culture-treated plates coated with 50 µg/ml poly-D-lysine (Sigma-Aldrich) and 150 µg/ml fibronectin (Akron Biotechnology). Cells were grown in smooth muscle differentiation medium for 7 days, as previously described (Ishii et al., 2012): DMEM (Gibco), 10% HI-FCS (Gemini Bio-Products), 100 U/ml penicillin and 100 µg/ml streptomycin (Corning). Medium was sterile filtered prior to use. Half the medium was exchanged the day after seeding, then the medium remained unchanged for the duration of the 7-day differentiation process.

#### Generation of adipocytes

Primary cranial or trunk NC cells were seeded at 50,000 cells/cm^2^ onto tissue culture-treated plates coated with 50 µg/ml poly-D-lysine (Sigma-Aldrich) and 150 µg/ml fibronectin (Akron Biotechnology). Cells were expanded in basal medium for 3 days to achieve 80-90% confluency, prior to culturing in Complete MesenCult™ Adipogenic Medium (Mouse; STEMCELL Technologies) for an additional 14 days. Half the medium was exchanged every 3 days.

#### Generation of melanocytes

Melanogenic differentiation was induced using a method adapted from those previously described (Maurer et al., 2007). Primary cranial or trunk NC cells were seeded at 20,000 cells/cm^2^ onto tissue culture-treated plates coated with 50 µg/ml poly-D-lysine (Sigma-Aldrich) and 150 µg/ml fibronectin (Akron Biotechnology). Cells were grown for 10 days in melanogenic differentiation medium: DMEM-low glucose, pyruvate (Gibco), 2% chick embryo extract (prepared as described in Stemple and Anderson, 1992), 10% HI-FCS (Gemini Bio-Products), 1% N2 supplement (Gibco), 2% B27 supplement (Gibco), 100 U/ml penicillin and 100 µg/ml streptomycin (Corning). Medium was sterile filtered and supplemented with 1 ng/ml recombinant human bFGF (R&D Systems), 10 ng/ml recombinant human IGF1 (Gibco) and 100 nM endothelin 3 (ET3; Sigma-Aldrich). Half the medium was exchanged every other day.

### Evaluation of differentiation

#### Immunocytochemistry

Neuronal, glial, smooth muscle and chondrogenic differentiation was assessed via immunocytochemistry. Cultures were fixed at specified time points with 4% paraformaldehyde (PFA) for 30 min at room temperature and washed three times with PBS prior to immunostaining. Cells were then washed with PBS with 0.1% Tween 20 (PBST), blocked in 5% normal goat serum/PBST for 1 h and incubated with primary antibodies overnight at 4°C. The following primary antibodies were used: anti-TUJ1 (1:500, STEMCELL Technologies, #60052); anti-HuC/D (1:1000, Invitrogen, #A-21271); anti-GFAP (1:1000, Sigma-Aldrich, #G3893); anti-αSMA (1:500, eBiosciences, #14-9760-80); and anti-Col2a1 (1:500, Abcam, #ab34712). After washing, cells were incubated with secondary antibodies conjugated to Alexa Fluor 546 (goat anti-mouse #A11030 and goat anti-rabbit #A11035, Invitrogen). Single-focal-plane images of EYFP and rhodamine signals in NC cultures were acquired with a Zeiss AxioZoom.V16 fluorescence stereomicroscope equipped with an Orca R2 CCD camera, a Zeiss ApoTome and appropriate filter sets. The objective (PlanNeoFluar Z 2.3x size) on the AxioZoom.V16 has a 0.57 numerical aperture (NA). Fluorescent images were pseudocolored, as indicated in figure legends, merged using ZEN 2011 software (Zeiss) and exported to TIFF format using AxioVisionSE64 rel 4.9.1. (Zeiss).

#### Oil Red O staining

Adipogenic differentiation was assessed by staining with Oil Red O (Humason, 1962; Thermo Fisher Scientific). Cultures were fixed with 4% PFA at room temperature for 30 min, washed three times with PBS, then incubated in 60% isopropanol for 2 min. Cultures were then incubated with a 3:2 solution of 30% Oil Red O dissolved in isopropanol:deionized water for 5 min at room temperature. After incubation, the cultures were washed with distilled water and imaged using light microscopy.

#### L-DOPA reaction assay

Melanogenic differentiation was assessed by 3,4-dihydroxy-L-phenylalanine (L-DOPA) reaction assay (Tsuchiyama et al., 2013). Briefly, primary cells were fixed with 4% PFA at room temperature for 30 min, washed three times with PBS, then incubated with 5 mM L-DOPA (Sigma-Aldrich) in PBS at 37°C for 8 h in the dark. After incubation, the cells were washed with distilled water and imaged using light microscopy.

#### Alcian Blue staining

Chondrogenic micromass cultures were grown in parallel and fixed at Day 0, 2, 4, 6, 8, 10 and 14 with 4% PFA for 30 min at room temperature and washed three times with PBS prior to incubation with a 0.1% solution of Alcian Blue 8GX (Sigma-Aldrich) dissolved in acidic ethanol (5% concentrated hydrochloric acid, 70% ethanol) overnight at 4°C. After incubation, cultures were destained in acidic ethanol, washed with distilled water and imaged using light microscopy.

#### Quantification of nodule number

Alcian Blue-stained cultures were imaged at lower magnification in order to obtain a representative field of view of the entire culturing surface of a well (2 cm^2^) in a four-well plate. Four images from each well were aligned and stitched using the open source Hugin software (http://www.hugin.sourceforge.net). Nodule number was determined at each time point in duplicate using the particle analysis tool in ImageJ (Schneider et al., 2012). The results from three independent cultures were used to calculate the average number of nodules at each time point.

#### Alcian Blue extraction

Cartilage-matrix accumulation was assessed by extracting specifically bound Alcian Blue dye from the micromass cultures, as previously described (Paulsen et al., 1988). Micromass cultures at each time point in duplicate were solubilized in 4 M guanidine hydrochloric acid, pH 5.8 overnight at 4°C. Following incubation, absorbance at 600 nm was measured using a spectrometer (Eppendorf BioPhotometer). Absorbance values were normalized against those measured at Day 0. The results from three independent cultures were used to determine the net amount of cartilage-matrix present at each time point.

### Statistical analyses

All statistical analyses and graphical representations for the data presented were computed using GraphPad Prism, Version 7.0b for Mac OS X (GraphPad Software; http://www.graphpad.com). Significant differences between groups were determined using either repeated measures one-way analysis of variance (ANOVA; Fig. 1) or two-way ANOVA (Figs 2 and 7), as indicated. Comparisons between means were carried out using Tukey's post hoc multiple comparison test. All results are reported as mean±s.e.m. For all experiments, *P*-values<0.05 were considered statistically significant.

## Supplementary Material

Supplementary information
