# Supplementary material for: Establishment of a murine culture system for modeling the temporal progression of cranial and trunk neural crest cell differentiation
Source: Dis Model Mech. 2018 Dec 12;11(12):dmm035097. doi: 10.1242/dmm.035097 (PMC6307900; doi:10.1242/dmm.035097)
Supplement: Supplementary information [file dmm-11-035097-s1.pdf]

SUPPLEMENTARY INFORMATION

Biological isolates		Cranial NC					Trunk NC				
Pregnant dams	Number of embryos	Total cells recovered	Cells per embryo	Doubling rate (hours)	Cells per embryo by P3	Total number of cells by P3	Total cells recovered	Cells per embryo	Doubling rate	Cells per embryo by P3	Total number of cells by P3
3	28	2.77E+05	9.89E+03	44.6	8.69E+05	2.43E+07	7.26E+04	2.59E+03	40.0	3.81E+05	1.07E+07
2	18	1.20E+05	6.64E+03	42.9	6.97E+05	1.25E+07	5.19E+04	2.88E+03	42.6	3.12E+05	5.62E+06
1	7	7.50E+04	1.07E+04	44.9	9.14E+05	6.40E+06	3.00E+04	4.29E+03	32.6	1.96E+06	1.37E+07
**average**	8.83		9.08E+03	44.1	8.37E+05	7.39E+06		3.25E+03	38.4	5.89E+05	5.20E+06

Table S1. Cell numbers and doubling rates for biological replicates. Each primary isolate consisted of cells pooled from 1-3 litters of embryos (7-28 embryos total) depending on how many females set up for breeding were carrying E9.5 embryos on the day of dissection. *Method:* "Doubling rate" was determined using an online doubling time calculator (http://www.doubling-time.com/compute.php). "Cells per embryo" was calculated by dividing the total number of cells recovered at isolation by the total number of embryos. "Cells per embryo by P3 (passage 3)" was calculated using the doubling rate formula (cells per embryo x $2^{\text{(hours in culture (288)/doubling rate)}}$). "Total number of cells by P3" for each experiment was calculated by multiplying "number of embryos" by "cells per embryo by P3". Average number of embryos per litter was calculated by dividing the total number of embryos recovered from the three biological replicates (53) by the total number of litters (6). Average "Cells per embryo" and "Doubling rate" were calculated by taking the average of the three biological replicates. Average "Cells per embryos by P3" was calculated using the doubling rate formula (average cells per embryo x $2^{\text{(hours in culture (288)/average doubling rate)}}$). Average "Total number of cells by P3" was calculated by multiplying "average number of embryos" by "average cells per embryo by P3".

Disease Models & Mechanisms • Supplementary information

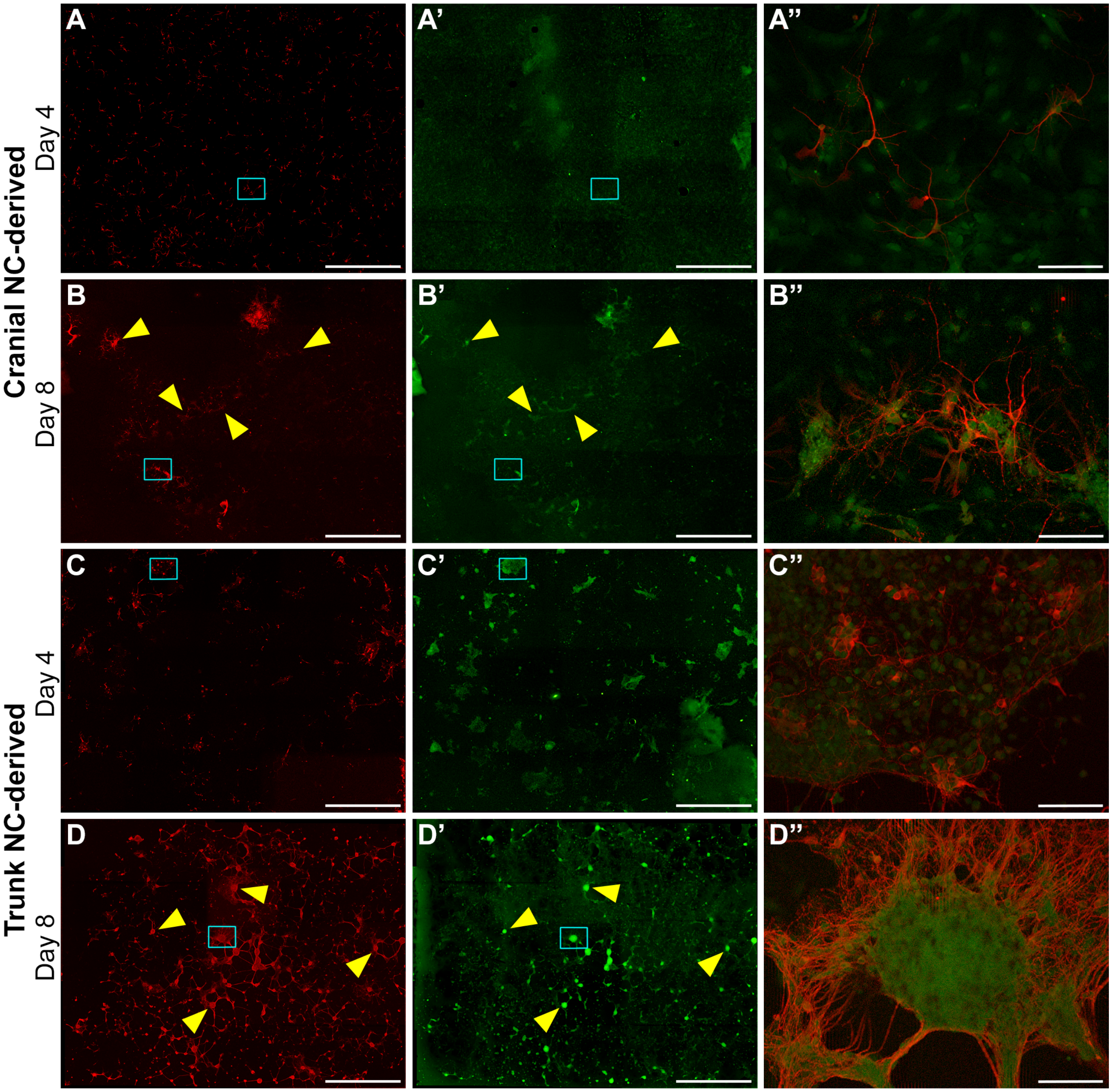

Figure S1. Wide field comparisons of neuronal differentiation in cultures derived from cranial and trunk NC. Neuronal differentiation of cranial- and trunk-derived NC cells was observed after 4 and 8 days in differentiation medium containing NT3 and NGF. Differentiation was assessed by immunostaining for TUJ1, a neuron-specific class III β-tubulin. In order to visualize the extent of differentiation within the cultures, we obtained overlapping, high resolution images spanning the entire culture well. **A-D:** TUJ1

Disease Models & Mechanisms • Supplementary information

immunostaining. **A'-D':** EYFP expression (lineage tracer in the NC cells). **A"-D":** Higher magnification, merged images of the boxed regions in A-D' (TUJ1, red; EYFP, green). Images were aligned and stitched using the open source Fiji software (Preibisch et al., 2009). Cells with neurites are observed throughout cultures from both cranial and trunk NC, however, aggregation of neurons is more prevalent in cultures derived from trunk NC at both time points. At day 4, individual neurons extending neurites were observed throughout the cranial NC culture (**A, A"**), while trunk NC-derived neurons are observed both singly and in small loosely formed aggregates (**C, C"**). By day 8, loose aggregates of cranial NC-derived neurons can also be observed (examples indicated by *arrowheads* and *boxed* region in **B,** and as magnified boxed region in **B"**), however, aggregates formed by trunk NC-derived neurons are more compact and well-defined with extensive neuritic outgrowth (examples indicated by *arrowheads* and *boxed* region in **D,** and magnified boxed region in **D"**). Scale bars: (A-D and A'-D') 2 mm; (A"-D") 100 µm.

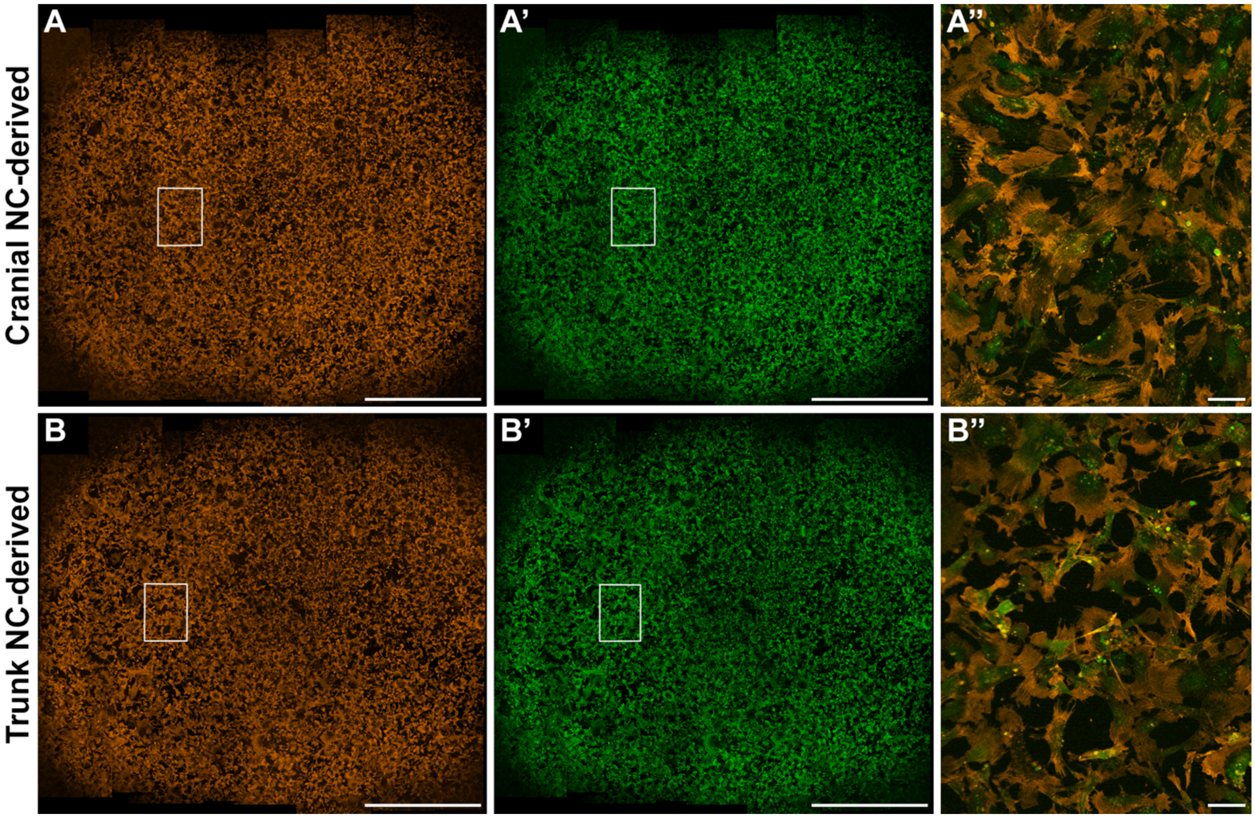

Figure S2. Wide field comparisons of smooth muscle differentiation in cultures derived from cranial and trunk NC. Smooth muscle differentiation of cranial- and trunk-derived NC cells was observed throughout the culture after 7 days in differentiation medium containing FCS. Differentiation was assessed by immunostaining for alpha smooth muscle actin (αSMA). In order to visualize the extent of differentiation within the cultures, we obtained overlapping, high resolution images spanning the entire culture well. **A, B:** αSMA immunostaining, **A', B':** EYFP expression (lineage tracer in the NC cells). Images were aligned and stitched using the open source Fiji software (Preibisch et al., 2009). **A", B":** Higher magnification, merged images of the boxed regions in A-B' (αSMA, orange; EYFP, green). Images were aligned, stitched and pseudocolored using the open source Fiji software (Preibisch et al., 2009). Scale bars: (A, B and A', B') 2 mm; (A", B") 100 µm.

Disease Models & Mechanisms • Supplementary information

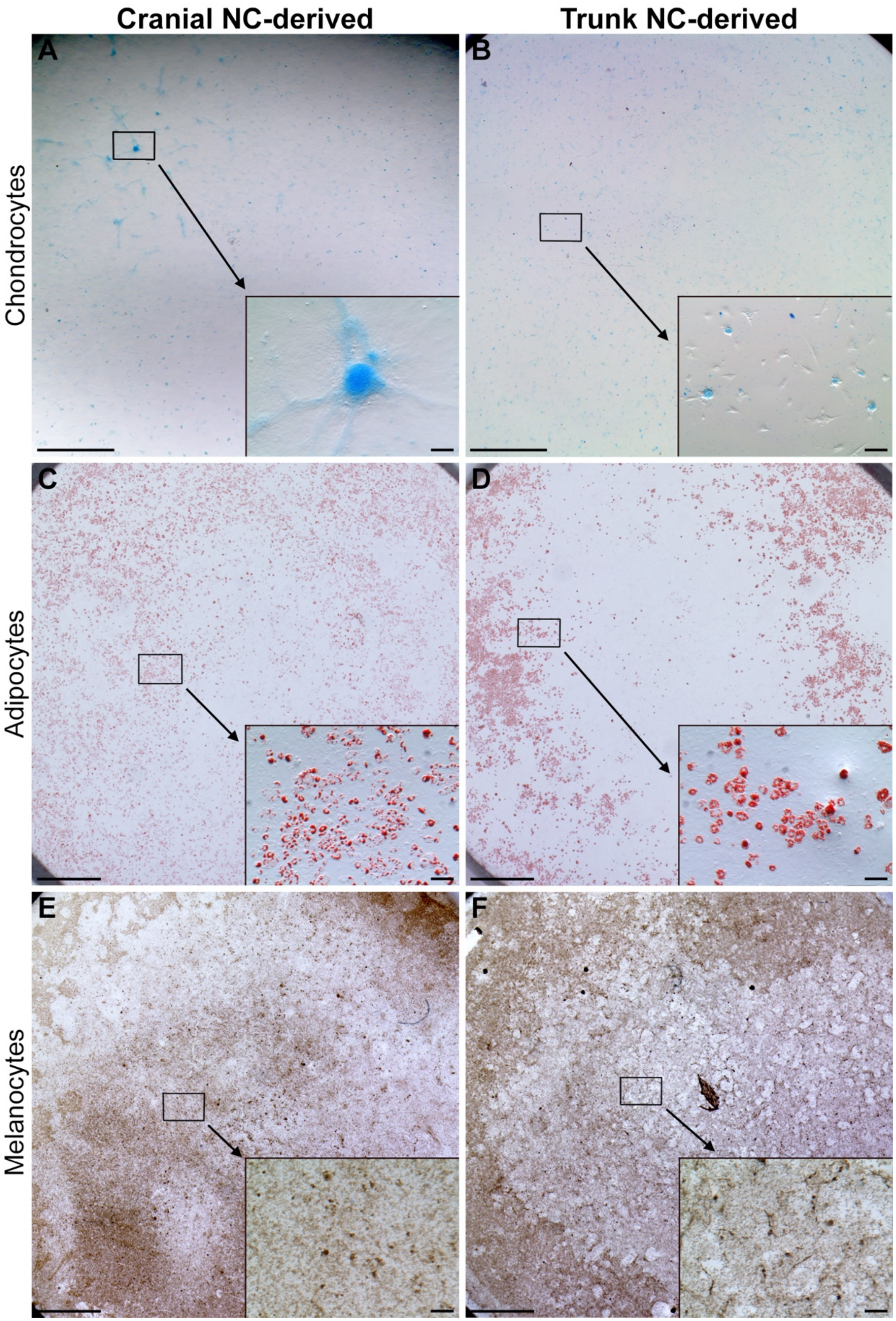

Figure S3. Wide field comparisons of chondrogenic, adipogenic, and melanogenic differentiation in cultures derived from cranial and trunk NC. Differentiation of

Disease Models & Mechanisms • Supplementary information

cranial- and trunk-derived NC cells into chondrocytes, adipocytes and melanocytes was observed throughout the cultures (**A, B:** chondrocytes (*blue*) detected by Alcian blue staining after 14 days in differentiation medium containing TGF-β3; **C, D:** adipocytes (*red*) detected by Oil red O staining after 14 days in Adipogenic Medium from STEMCELL Technologies; and **E, F:** melanocytes (*brown*) detected based on tyrosinase activity after 10 days in differentiation medium containing ET3). In order to visualize the extent of differentiation within the cultures, we obtained overlapping, high resolution images spanning the entire culture well. Images were aligned and stitched using the open source Hugin software (http://www.hugin.sourceforge.net). Higher magnification images of the boxed regions are shown in the insets. Scale bars: 2 mm; insets, 100 μm.

Disease Models & Mechanisms • Supplementary information

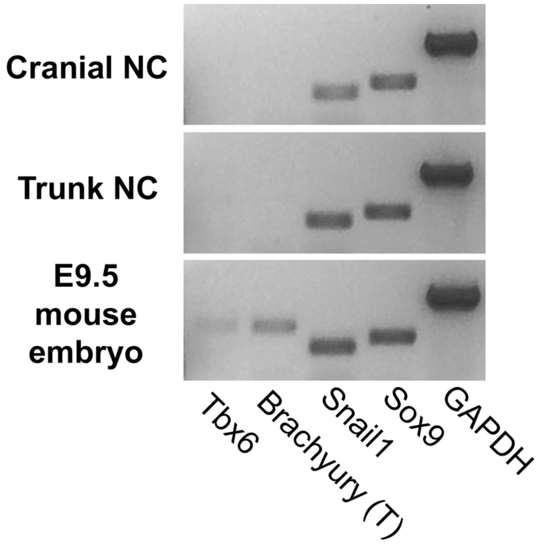

Figure S4. Mesodermal markers are not detected in cultured cranial and trunk NC cells. Cultured NC cells were compared with whole E9.5 embryos for the expression of a pan-mesodermal marker, brachyury (*T*), or a marker for axial mesoderm, *Tbx6*, using reverse transcriptase PCR. NC markers, *Snail1* and *Sox9*, and housekeeping gene, *Gapdh*, served as positive controls. A representative gel is shown; however, identical results were obtained from three independent biological samples. *Method:* PCR was carried out on cDNA prepared from RNA isolated from cultured cranial or trunk NC after three passages, or from whole E9.5 mouse embryos as described in the methods. PCR primers: *Tbx6* (5'- TGAAGATCGCAGCCAATCCC-3', 5'-TGAAAAGCGGCAGGGTGTAG-3'); *Brachyury (T)* (5'-CCAGCTCTAAGGAACCACCG-3', 5'- AAAGAACTGAGCTCCCAGCC-3'); *Snail1* (5'-CTGCACGACCTGTGGAAAG-3', 5'-GCCTGGCACTGGTATCTCTT-3'); *Sox9* (5'-AGTCGGTGAAGAACGGACAA-3', 5'-CCCTCTCGCTTCAGATCAACT-3'); *GAPDH* (5'-GCTCATGACCACAGTCCATGC-3', 5'-GTTGGGATAGGGCCTCTCTTG-3').

Disease Models & Mechanisms • Supplementary information

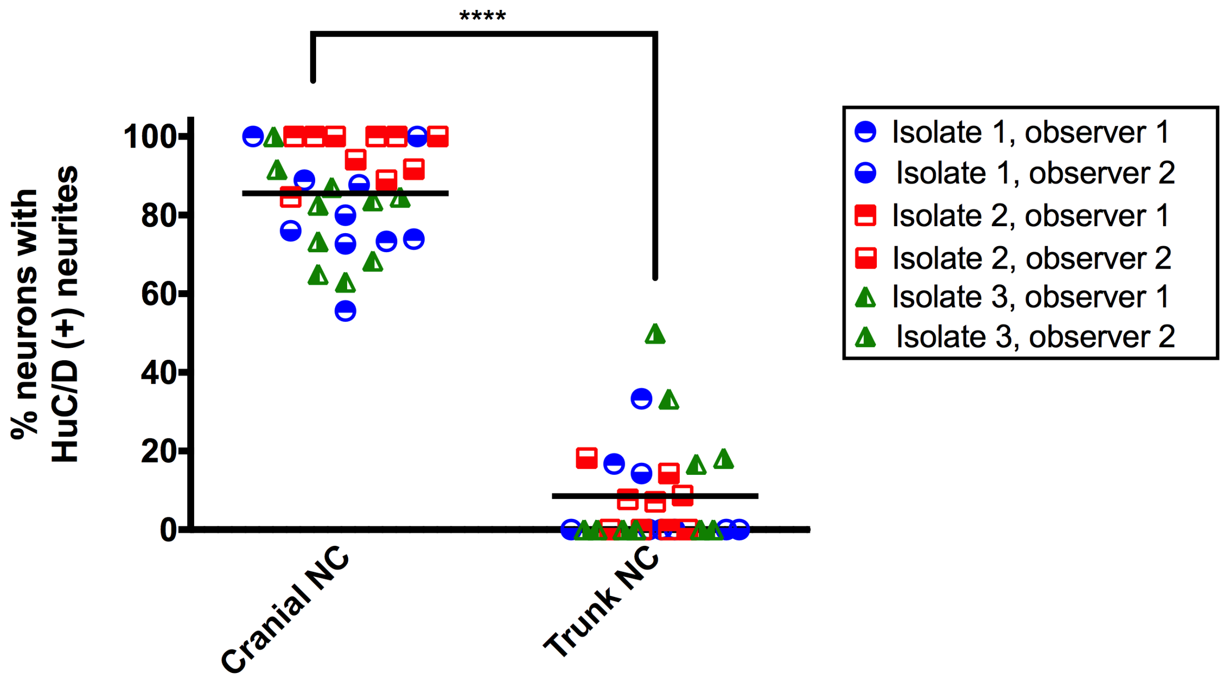

Figure S5. Neuritic expression of HuC/D is more prevalent in neurons derived from cranial NC when compared to those derived from trunk NC. *Method:* Images of neurons differentiated from cranial and trunk-derived NC and subjected to HuC/D immunofluorescence staining for experiments described in Fig. 5 were quantified with respect to the percentage of neurons extending HuC/D positive neurites. Five images were obtained from differentiated cultures in each biological replicate. Cells with neurites were identified in the green channel (EYFP). These cells were then assessed for HuC/D expression in the red channel to calculate the percentage of neurons with HuC/D in processes. Each image was analyzed separately by two blinded observers, and both sets of observations are displayed in the graph. The Grubbs test was used to eliminate outliers in the cranial and trunk data sets from each observer, resulting in elimination of two points: (1) trunk, isolate 2, observer 1 and (2) trunk, isolate 1, observer 2. Two-way ANOVA analysis with Tukey's multiple testing correction determined no significant effects of observer or isolate, but a significant effect between cranial and trunk NC ($p<0.0001$). Each dot represents one observation of one image and is color-coded by biological replicate and observer as indicated. Black horizontal line indicates grand mean across replicates.

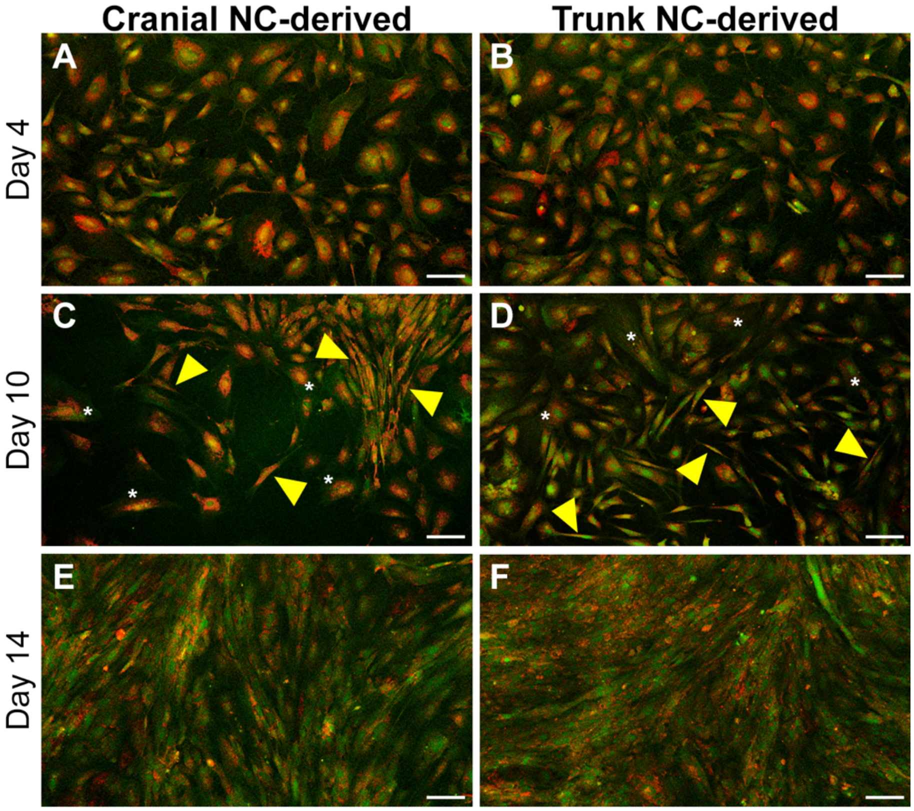

Figure S6. Primary cranial and trunk NC cells express Schwann cell marker, ErbB3, during glial differentiation . Schwann cell differentiation of cranial- and trunk-derived NC cells was observed after 4, 10 or 14 days in differentiation medium containing BMP2 and LIF. Differentiation was assessed by immunostaining for ErbB3, a receptor tyrosine kinase enriched in Schwann cells. ErbB3-positive cells were observed in both cranial and trunk NC cell populations at Day 4 **(A, B)**. By Day 10, some of the ErbB3-positive cells extended flattened, sheet-like processes **(C, D**; *asterisks***)** while the other ErbB3-positive cells displayed an elongated, spindle-like morphology **(C, D**; *arrowheads***)**. After 14 days, most ErbB3-positive cells in both populations exhibited an elongated, bipolar morphology and cells extending flattened, sheet-like processes were only occasionally observed **(E, F)**. Phenotypic characteristics of the cells at each time point were consistently observed (n=6; duplicate cultures from each of three independent cell isolates). All cells are derived from *Sox9cre; R26R-EYFP* mice and express EYFP (*green*). Red staining: ErbB3. Scale bars: 100μm.

References

Preibisch, S., Saalfeld, S. and Tomancak, P. (2009). Globally optimal stitching of tiled 3D microscopic image acquisitions. *Bioinformatics* **25**, 1463-5.
